# The development of dissolved oxygen forecast model using hybrid machine learning algorithm with hydro-meteorological variables

**DOI:** 10.1007/s11356-022-22601-z

**Published:** 2022-09-01

**Authors:** Abul Abrar Masrur Ahmed, S. Janifer Jabin Jui, Mohammad Aktarul Islam Chowdhury, Oli Ahmed, Ambica Sutradha

**Affiliations:** 1 Department of Infrastructure Engineering, University of Melbourne, Parkville, VIC 3010 Australia; 2 School of Mathematics Physics and Computing, University of Southern Queensland, Springfield, QLD 4300 Australia; 3 Department of Civil and Environmental Engineering, Shahjalal University of Science and Technology, 3114 Sylhet, Bangladesh; 4 School of Modern Sciences, Leading University, Sylhet, 3112 Bangladesh

**Keywords:** Dissolved oxygen, Forecasting, Hybrid model, MARS, MODWT, Surma River, Bangladesh

## Abstract

Dissolved oxygen (DO) forecasting is essential for aquatic managers responsible for maintaining ecosystem health and the management of water bodies affected by water quality parameters. This paper aims to forecast dissolved oxygen (DO) concentration using a multivariate adaptive regression spline (MARS) hybrid model coupled with maximum overlap discrete wavelet transformation (MODWT) as a feature decomposition approach for Surma River water using a set of water quality hydro-meteorological variables. The proposed hybrid model is compared with numerous machine learning methods, namely Bayesian ridge regression (BNR), k-nearest neighbourhood (KNN), kernel ridge regression (KRR), random forest (RF), and support vector regression (SVR). The investigational results show that the proposed model of MODWT-MARS has a better prediction than the comparing benchmark models and individual standalone counter parts. The result shows that the hybrid algorithms (i.e. MODWT-MARS) outperformed the other models (*r* = 0.981, WI = 0.990, RMAE = 2.47%, and MAE = 0.089). This hybrid method may serve to forecast water quality variables with fewer predictor variables.

**Supplementary Information:**

The online version contains supplementary material available at 10.1007/s11356-022-22601-z.

## Introduction

The deterioration of the quality of water sources throughout the world is considered a wide-reaching issue of importance. Because of the rapid rise of communities and the diversity of their activities, this deterioration is speeding up, and it could constitute a severe threat to the aquatic environment and human health (Henderson et al. 2009; Hur and Cho 2012; Mouri et al. 2011; Su et al. 2011).

The dissolved oxygen (DO) in water is a critical water quality variable that is crucial for the proper functioning of the aquatic ecosystem (Ranković et al. 2010). DO demonstrate the water pollution in rivers (Heddam and Kisi 2018; Mohan and Kumar 2016) and the state of the river’s ecosystems (Mellios et al. 2015; Ranković et al. 2010). The concentration of dissolved oxygen (DO) in aquatic systems refers to the metabolism of the aquatic systems, and it reflects the transient balance between the oxygen system and the metabolic activity. The concentration of DO is affected by a variety of parameters, including salinity, temperatures, and pressure (US-Geological-Survey 2016). Researchers investigated the concentration and change of DO over the last decade since the dynamics of DO are nonlinear (Kisi et al. 2020). It is very desirable for water resource managers to develop a DO model for rivers that can reliably quantify and predict DO concentrations based on hydro-meteorological variables.

There are various methods available for estimating the DO concentration, but most of them are time-consuming and expensive to use since they require numerous parameters that are not readily available in most cases (Suen and Eheart 2003). More to the point, conventional data processing techniques are no longer appropriate for water quality modelling, which may be linked to the explanation that many parameters affecting water quality have a complicated nonlinear interaction with one another (Ahmed 2017; Xiang et al. 2006). There are specific issues in developing a water quality model for tiny streams or rivers due to the lack of available data, investment, and many different inputs to consider. As a result, certain well-known water quality analysis models, such as the United States Environmental Protection Agency (USEPA): QUAL2E and QUAL2K, WASP6, require a great deal of information that is not always readily available (Ahmed 2017). Moreover, these models are complex and sensitive and, therefore, tough to recognise.

Machine learning-based data-driven algorithms have become potentially widespread in the field of water quality modelling (Ahmed 2017; Ahmed and Shah 2017a; Forough et al. 2019; Kuo et al. 2004; Tomic et al. 2018) and hydrological modelling (Ahmed et al. 2021b, c; Ahmed and Shah 2017b; Yaseen et al. 2016). In addition, a number of artificial intelligence (AI)-based models for predicting and estimating DO concentrations have been developed such as soft computing (Tao et al. 2019), artificial neural networks (ANNs), and hybrid ANN (Keshtegar et al. 2019; Zounemat-Kermani et al. 2019), fuzzy-based models (Heddam 2017; Raheli et al. 2017), multivariate adaptive regression spline (MARS) (Rezaie-Balf et al. 2019), support vector machine (SVM) (Heddam and Kisi 2018; Li et al. 2017b), extreme learning machine (ELM) (Heddam 2016; Heddam and Kisi 2017; Zhu and Heddam 2020), quantile regression (Ahmed and Lin 2021), and other potential approaches were applied for dissolved oxygen concentration modelling.

This study investigates the utilisation of multivariate adaptive regression splines (MARS) (Friedman 1991) to describe DO dynamics’ intrinsic nonlinear and multidisciplinary relationship. Like neural networks, no prior information on the numerical function is required for MARS. The benefit of the MARS model is that it can accomplish complex data by grouping related data collected, permitting it to understand easily (Zhang and Goh 2016). Considering the positive attribute, the MARS model has been used in hydrology (Deo et al. 2017b; Heddam and Kisi 2018; Kisi and Parmar 2016; Yin et al. 2018) and the energy sector (Al-Musaylh et al. 2019). Heddam and Kisi (2018) applied the least-square support vector machine (LSSVM), multivariate adaptive regression splines, and M5 model tree (M5T) for daily dissolved oxygen forecasting. The authors found the MARS model a substantial forecasting approach with a limited number of predictor variables. Therefore, incorporating the hybrid approaches and a potential feature selection algorithm may boost the result of forecasting. Nevertheless, the hybrid MARS models are yet to be executed in the study sites of Bangladesh.

Using multi-resolution analysis (MRA), a technique for extracting data features, the prediction performance can be enhanced significantly. Using the EMD, you can decompose a signal following the spirit of the Fourier series into a specific number of components. A coefficient representing Gaussian white noise with a unit variance is introduced sequentially to the time series in CEEMDAN-based decomposition to reduce the complexity and avoid the intricacy of the time series (Prasad et al. 2018). A coefficient denoting Gaussian white noise with covariance matrices is introduced sequentially to the time series in CEEMDAN-based decomposition to reduce the complexity and prevent the intricacy of the time series (Di et al. 2014). Previous studies have used CEEMDAN in forecasting soil moisture (Ahmed et al. 2021a; Prasad et al. 2018, 2019) with an earlier version (i.e. EEMD) used in forecasting stream-flow (Seo and Kim 2016) and rainfall (Beltrán-Castro et al. 2013; Jiao et al. 2016; Ouyang et al. 2016). Discrete wavelets transform (DWT) has been employed (Deo and Sahin 2016; Deo et al. 2016; Nourani et al. 2014, 2009) in different fields of hydrology. On the other hand, DWT has a limitation that prevents it from extracting all the features of the predictors in its entirety. An enhanced discrete wavelet transforms (DWT), such as the MODWT, can solve these problems (Cornish et al. 2006; Prasad et al. 2017; Rathinasamy et al. 2014). Al-Musaylh et al. (2020) successfully used MODWT to decompose the short-term electricity demand of Australia. The study incorporated the MODWT by separately splitting the data to training, testing, and validation to calculate the detailed approximation, as Quilty and Adamowski (2018) prescribed. The potential application of MODWT is further approved by Prasad et al. (2017), where MODWT was used to forecast stream-flow. However, neither the MODWT nor the DWT decomposition model has incorporated the MARS model in DO forecasting, as attempted in this study.

The feature selection technique, namely neighbourhood component analysis (NCA) for regression, was used in this investigation. As a result of the algorithm being slowed down by the extraneous and redundant features, the prediction model is less accurate (Arhami et al. 2013) different feature selection methods have been utilised in predictive models (Ahmed et al. 2021a; Prasad et al. 2017, 2019). The NCA method has been successfully applied by Ahmed et al. (2021a) to forecast surface soil moisture. The study demonstrates that the feature weight calculated by NCA was found successful in forecasting soil moisture and to the study by Ghimire et al. (2019b), where they applied NCA for solar radiation forecasting. Forecasting DO concentration with a machine learning method incorporated with the NCA feature selection method and feature decomposition methods would substantially increase forecasting performance.

To the author’s knowledge, there has been no systematic comparison of various feature decomposition strategies in improving MARS performance for daily DO estimates. The fundamental contribution of this study is the selection of an appropriate feature decomposition algorithm (i.e. MODWT, DWT, CEEMDAN, EEMD, and EMD) tailored MARS model for DO prediction. While effective adjustment of MARS parameters via feature decomposition algorithms can increase prediction accuracy, the incorporation of feature selection and feature decomposition theories can aid decision-makers in making the optimal choice for the best prediction model. Because attempting all available optimisation techniques is practically impossible, the scope of the current study has been reduced to a few potential algorithms to be merged with the MARS. As a result, the goal of this study is to (1) use 5 feature decomposition techniques to modify MARS ability, (2) compare the performances of hybridised MARS models, and (3) rank the hybridised MARS models using hydro-meteorological variables. The findings of this work will be a helpful tool that can provide valuable information for better water management.

## Materials and methods

### Theoretical frameworks of proposed models

#### Multivariate adaptive regression spline

According to Friedman (1991), a non-parametric and nonlinear regression technique, the multivariate adaptive regression spline (MARS), was utilised in this investigation. MARS uses numerous splines to build nodes between these lines (Friedman 1991). The underlying functional link between inputs and outputs is not assumed in the MARS model. The data in each spline is assigned using basis functions (BF) in MARS models. It is possible to express the BF as a single equation between two knots. Two adjacent data domains converge at a knot, and the output is continuous. An adaptive regression algorithm is used (Heddam and Kisi 2018). The MARS model depicts the piecewise relationship between the input and output variables using numerous lines. The over-fitting of training data is avoided by setting a predefined minimum number of observations between knots (Heddam and Kisi 2018).

Let y be the target output, and a matrix of n input variables be the vector x = (${x}_{1},\ldots ,{x}_{n})$. The data are then presumed to be created from an undisclosed ‘true’ model. In the case of a straight answer, this will be as follows:

1 $$y=f({x}_{1},\ldots .,{x}_{n})+\xi =f(x)+\xi$$

In which $\xi$ is the distribution of the model error, and n is the number of training data points. By adding sufficient BFs, MARS approximates the f(*.*). For linear functions piecewise: max (*0, x-t*) where a knot exists at position *t* (Zhang and Goh 2016). The max (.) equation implies that only the positive portion of (.) is used; otherwise, a zero value will be given corresponding to:

2 $$\mathrm{max }(0,\mathrm{ x}-\mathrm{t})=\left\{\begin{array}{c}x-t, if x\ge t\\ 0, otherwise\end{array}\right.$$

Thus, *f* (x) is constructed as a linear BF(x) combination:

3 $$f(\mathrm{x}) = {\beta }_{0}+\sum_{i=1}^{n}{\beta }_{i}BF(x)$$

The coefficients $\beta$ are constants, calculated using the form of least squares. Initially, *f* (x) is applied to input data in a forward–backward stepwise process to determine the knot’s position where the feature value varies (Deo et al. 2017b). A broad model is built at the end of the forwards’ stage to over-fit the qualified input data. According to the generalised cross-validation, the model is optimised by deleting one last basis function from the model (GCV). GCV for a model is computed as follows for the training data with *n* observations:

4 $$\mathrm{GCV}=\frac{\frac{1}{n}{\sum }_{i=1}^{n}{\left[{y}_{i}-f({x}_{i})\right]}^{2}}{{\left[1-\frac{M+d\times (M-1)/2}{n}\right]}^{2}}$$

where *M* is the number of BF, *d* is the penalising parameter, *n* is the number of measurements, and *f* ${(x}_{i})$ denotes the MARS model’s expected values.

MARS is a non-parametric regression modelling technique that is flexible and does not make any assumptions about the relationships between the variables (Stull et al. 2014). The model is simple to understand and interpret (Kuhn and Johnson 2013). MARS models typically exhibit a favourable bias-variance trade-off. While the models are sufficiently flexible to account for nonlinearity and variable interactions (and so have a relatively low bias), the limited nature of the MARS basis functions precludes excessive flexibility (thus, MARS models have relatively low variance).

#### Maximal overlap discrete wavelet transforms

Distinctive wavelet transforms (DWTs) are modified by the maximal overlap discrete wavelet transform (MODWT) (Li et al. 2017a). Ideally, time series analysis can be done using the MODWT’s appealing qualities, which prevent missing data without subsampling. MODWT’s ability to extract additional information is enhanced because the coefficients of decomposed components in each layer are identical to the original time series. Time-series data are broken down into high-pass and low-pass filters using MODWT, which handles two feature sets. Further, high-pass filters can be broken down into several information levels depending on the suitable time frame (He et al. 2017). Low-pass filters reflect the real-time-series signal pattern called an approximation. The signal ${\varkappa }_{m}$ is decomposed through wavelet low-pass ${\i}_{m}$ and high-pass detail filters ${h}_{m}$ and reconstructed by digital reconstruction filters complementing decomposition filters. This principle is described in the equations below:

5 $${\mathcal{X}}_{m+1}\left(K\right)= {\sum}_{p}{h}_{p-2k}{\mathcal{X}}_{m}(P)$$

6 $${d}_{m+1}\left(K\right)= {\sum}_{p}{l}_{p-2k}{\mathcal{X}}_{m}(P)$$

7 $${\mathcal{X}}_{m}\left(K\right)= {\sum}_{p}{{h}^{^{\prime}}}_{p-2k}{\mathcal{X}}_{m+1}(P)+ {\sum}_{p}{{l}^{^{\prime}}}_{p-2k}{\mathcal{X}}_{m+1}(P)$$

### Comparing models

In this study, we proposed a MODWT-MARS model to predict the dissolved oxygen of a running river. To find a practical approach to machine learning methods and feature decomposition methods, a pool of six machine learning models and five feature decomposition methods were also incorporated. The theoretical description of the proposed algorithms (i.e. MODWT and MARS) was explained in the previous section, and this section provides a short overview of the comparing algorithms.

Breiman (2001) proposed an algorithm based on a random forest (RF), which included methods for regression and classification. The bootstrap resampling procedure generates a new set of training data from the initial training sample set *N*, and then bootstrap-set random forests are built using *K* decision trees. The RF model’s full specifications may be read here (Ali et al. 2020a). The random forests approach has become a prominent tool for classification, prediction, investigating variable relevance, selection, and outlier identification. RF comprises a group (ensemble) of basic tree predictors. Each tree may generate a response given a collection of predictor values (Jui et al. 2022; Yu et al. 2017).

With regularisation and the kernel technique, it is possible to reduce over-fitting using the KRR (Kernel Ridge Regression) regression model (Saunders et al. 1998). The “kernel technique” can be used to generate a nonlinear form of ridge regression. Extending the general framework, kernel ridge regression allows nonlinear prediction. Linear, polynomial and Gaussian kernels are only some of the many options available for enhancing overall performance (You et al. 2018). The suggested KRR technique has the fundamental advantage of learning a global function and predicting any target variable using a regularised variation of least squares.

The Bayesian modelling approach uses hierarchical data (Huang and Abdel-Aty 2010). Bayesian regression uses this regularisation parameter, easily tailored to the data. The Gaussian maximum posterior estimate is discovered before the coefficient *w* and, with an accuracy of λ (-1), is treated as a random variable instead of a lambda. In contrast, most decision-making analyses based on maximum likelihood estimation entail determining the values of parameters that may significantly impact the analysis outcome and for which there is considerable uncertainty. The capacity to include previous information is one of the primary advantages of the Bayesian technique (Saqib 2021).

A machine learning kernel method known as SVR (Support Vector Regression) can be used for various purposes, including forecasting time series. SVRs that use kernels can also learn the nonlinear trend of the training data. There are three SVR models to pick from, each with a different kernel (RBF, poly, and linear) (Yang et al. 2017). It should also be noted that the proposed KRR model in its generic sense has been used in many research including the forecasting of precipitation (Ali et al. 2020b), drought (Ali et al. 2019), wind speed (Alalami et al. 2019; Douak et al. 2013; Mishra et al. 2019; Naik et al. 2018; Zhang et al. 2019), and solar power (Dash et al. 2020).

K-nearest neighbours (KNN) algorithm is implemented using instance-based learning, which serves two purposes: (1) estimating the test data density function and (2) categorising the test data obtained from the test patterns (Shabani et al. 2020). Choosing the number of neighbours (k) is a crucial stage. This method’s efficiency depends on selecting samples from the nearest reference database (or most similar). If *k* is significant, other points from other classes can be placed inside the desired range of possibilities (Wu et al. 2008). The KNN method has been successfully applied previously (Ghiassi et al. 2017; Liu et al. 2020).

This study incorporated five decomposition methods (i.e. DWT, EMD, EEMD, MODWT and CEEMDAN) and six machine learning methods (i.e. MARS, RF, BNR, SVR, KNN and KRR) to address the prediction problem of dissolved oxygen concentration. Hyperspectral feature decomposition is DWT-assisted, and the features are evaluated for their efficacy in discriminating between subtly different ground covers (Bruce et al. 2002). The theoretical explanation of the method is explained by other researchers (Agbinya 1996; Fowler 2005; Shensa 1992). Most recently, Huang et al. (Huang et al. 1998) developed an empirical mode decomposition (EMD) method for analysing the information contained in data derived from non-stationary and nonlinear systems. This algorithm decomposes the signal into a series of oscillatory functions that are ‘well-behaved,’ which are referred to as the intrinsic mode functions in this context (IMFs). When used with the powerful adaptive EMD tool, it behaves as a dyadic filter bank (Flandrin et al. 2004). It is handy for filtering out noise in the measurement domains (Khaldi et al. 2008). Torres et al. (2011) implemented the CEEMDAN process to reduce the computational cost and retain the ability to eliminate mode mixing. The readers are requested to go through the previous studies (Ahmed et al. 2021a; Zhang et al. 2017; Zhou et al. 2019) for getting further information on CEEMDAN.

### Study area and data

**Fig. 1:**
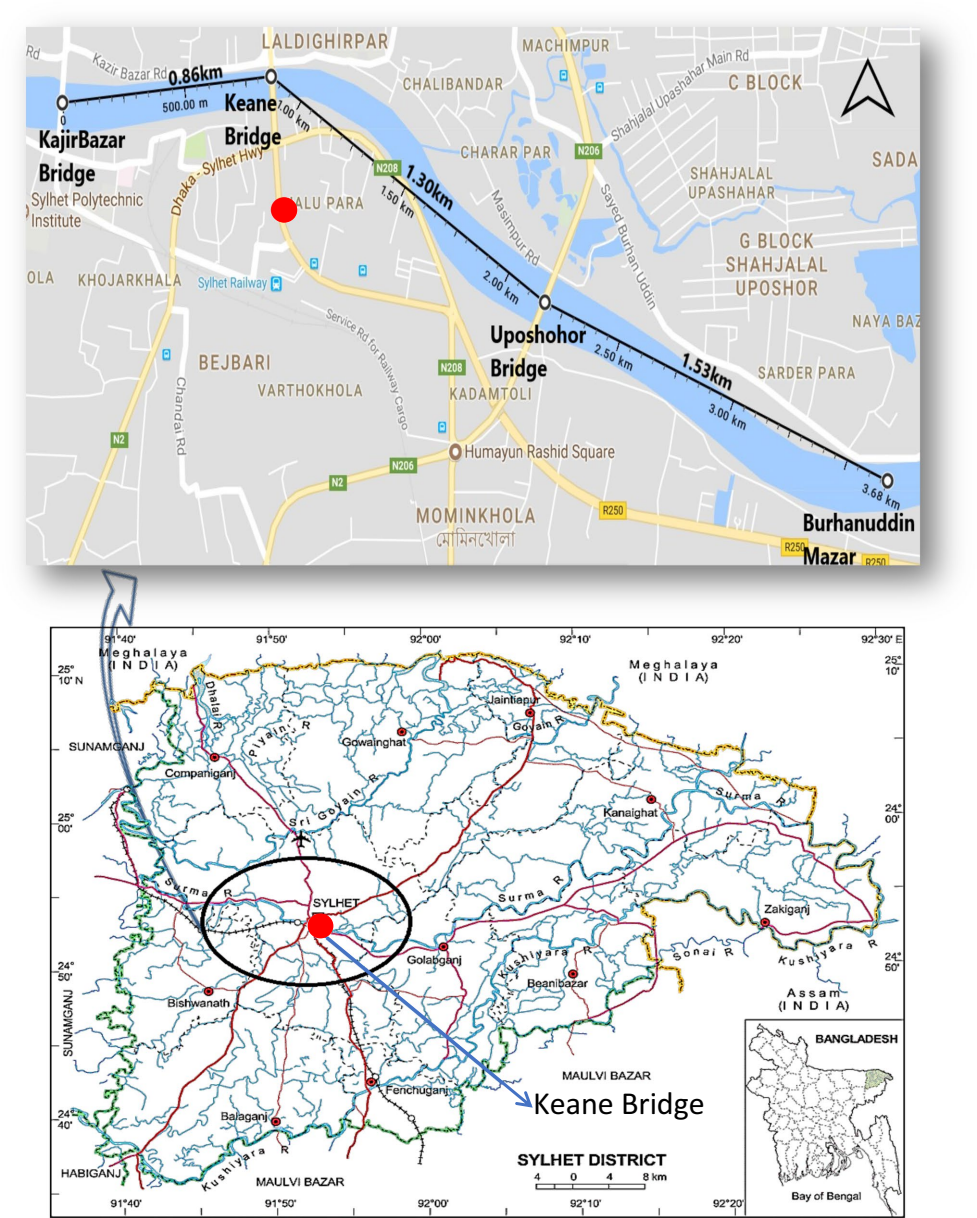
The study region showing the Keane Bridge station of Surma River, Sylhet, Bangladesh

The Surma River, Bangladesh, provided daily water quality factors. Figure 1 depicts the Surma River monitoring stations. This river drains one of the heaviest runoffs in the Surma-Meghna Basin system (Chowdhury and Ali 2006). The Surma River originates in Assam’s Cachar district, flows through Bangladesh’s Sylhet and Sunamganj districts, joins the Meghna River near Bhairab Bazar Kishoreganj, and empties into the Bay of Bengal. Many studies are found regarding water quality analysis (Ahmed 2017; Ahmed and Shah 2017a, b), riverbank erosion (Islam and Hoque 2014), stream flows (Ahmed and Shah 2017b), and water level modelling (Biswas et al. 2009). The Surma River’s Keane Bridge station provided the study’s water quality variables between January 2017 and December 2019 obtained 15 cm to 20 cm below the surface.

The selection of prospective predictive factors is critical for predictive modelling. Various studies reveal that some variables predict DO better than the others (Ahmed 2017; Tomic et al. 2018). Ahmed (2017) used Biological Oxygen Demand (BOD) and Chemical oxygen demand (COD) for predicting the dissolved oxygen of the Surma River. Kisi and Ay (2012) observed that the temperature, pH, and electrical conductivity are highly influential over Fountain Creek, Colorado. However, Ranković et al. (2010) claimed that pH and water temperature have a practical relation in DO prediction, whereas nitrates, chloride, and total phosphate have poor connections. It is found that pH is a standard variable for predicting DO values using ANN, followed by temperature. However, along with pH and temperature, some authors used oxygen-containing (PO_4_^3−^, NO_3_-N) variables or oxygen demanding variables (NH^−4^ N, COD, and BOD) (Wen et al. 2013). Turbidity (Iglesias et al. 2014) and total solid can be considered essential water quality parameters, as their high value indicates typically high values of other parameters associated with water quality. The missing values were interpolated from two adjacent values. The fundamental statistics of the input variables are tabulated in Table 1.

**Table 1:** Basic statistics i.e. minimum (min), maximum (max), mean (M), standard deviation (SD), and coefficient of variation (CV) of the water quality variables in Surma River, Sylhet, Bangladesh

Variable	Acronyms	Unit	Min	Max	Mean	SD	CV (%)
Humidity	h	%	0.01	3.79	0.53	0.70	132
Water temperature	w	^0^C	0.18	4.0	1.53	1.05	69
Rainfall	r	mm	8.00	127	32.66	20.99	64
TDS	td	Mg/l	10.0	522	142.3	102.15	72
pH	p	-	5.70	8.25	6.92	0.55	8
Turbidity	tr	(NTU)	4.18	42.62	11.84	7.37	62
Air temperature	a	^0^C	12.30	33.30	27.10	4.93	20.00
DO	d	(mg/l)	1.90	17.30	5.40	2.45	45

### Development of MODWT-MARS model

**Fig. 2:**
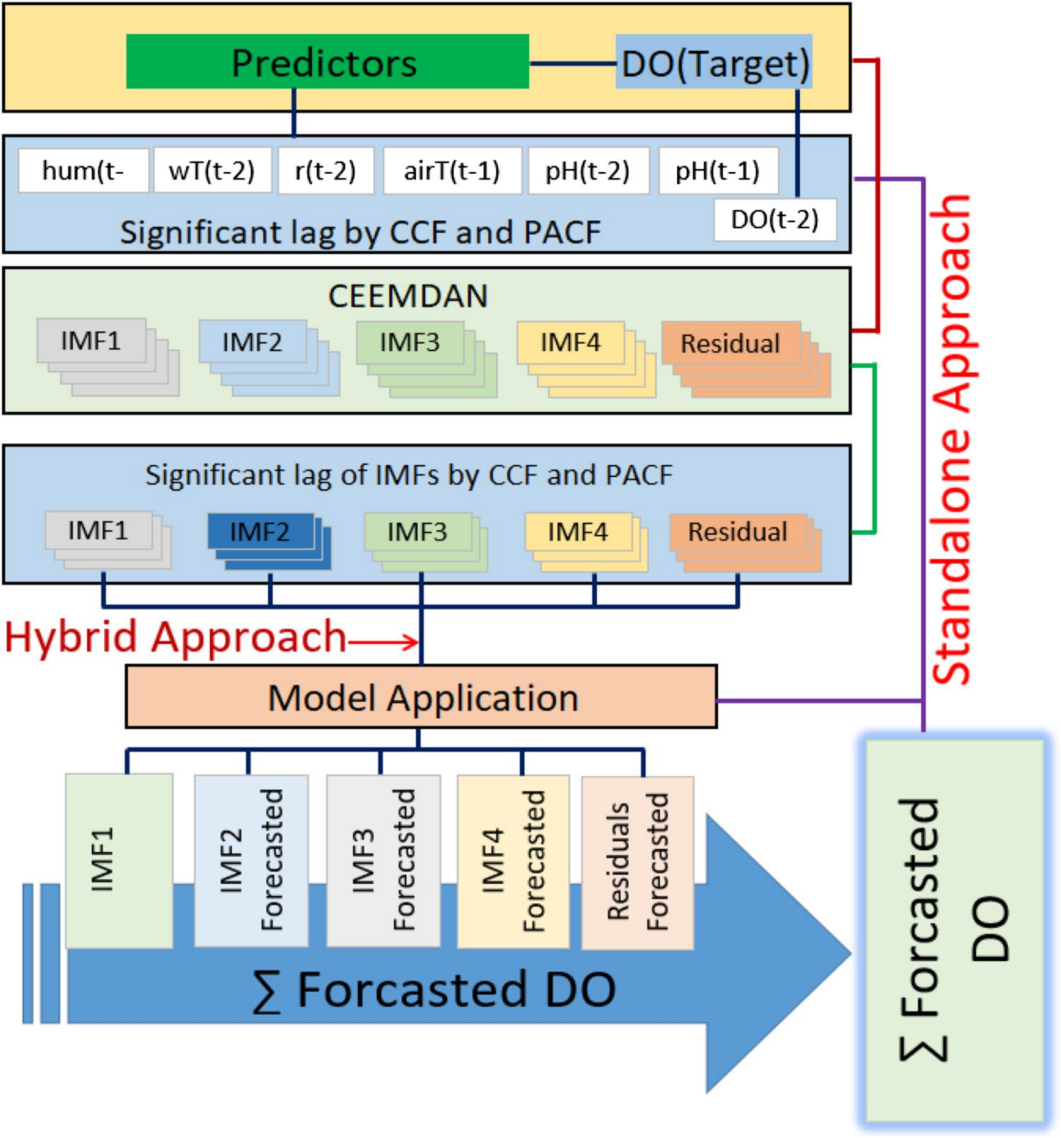
The study's workflow details the steps in the model designing phase and the proposed hybrid CEEMDAN-MARS predictive models. Note: IMF = Intrinsic Mode Function, CCF = Cross-Correlation Functions, PACF = partial autocorrelation function, CEEMDAN = complete ensemble empirical mode decomposition with adaptive noise and DO = Dissolved Oxygen (mg/l)

The multi-phase MODWT-MARS model and other benchmark models were created in Python using the sci-kit-learn machine learning platform (Pedregosa et al. 2011b). All simulations were performed on a machine with an Intel i7 processor running at 3.6 GHz and 16 GB of RAM. Furthermore, a software platform such as ‘MATLAB2020’ is employed for feature selection using neighbourhood component analysis (NCA). However, tools such as *matplotlib* (Barrett et al. 2004) and *seaborn* (Waskom et al. 2020) are employed to visualise the forecasted DO. Figure 2 depicts the workflow of the proposed MODWT-MARS model.

**Fig. 3:**
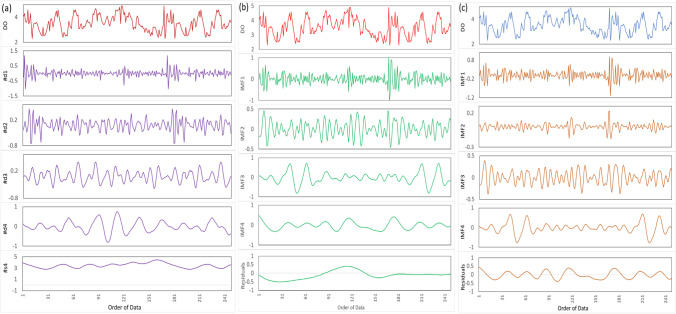
Time series of the **a** maximum overlap discrete wavelet coefficient (MODWC) of Dissolved Oxygen using MODWT, and intrinsic mode functions (IMFs) and the residual components after decomposing the DO in the training period using **b** CEEMDAN and **c** EEMD. The time series of the actual DO is plotted at the top of the figure

The wavelet transformation using MODWT was combined with the predictor variables filtered by the NCA approach to create the MODWT-MARS model. Identifying the wavelet-scaling filter types and decomposition level is vital in creating a substantial wavelet transformation model. Because there is no one approach to choose the optimal filter, Al-Musaylh et al. (2020) used a trial and error strategy. Quilty and Adamowski (2018) discovered an issue in the forecast model inputs due to erroneous wavelet decomposition during the wavelet-based forecasting model. The inaccuracy can be traced back to the decomposition process’s boundary conditions. They identified three problems: (1) improper use of future data, (2) unsuitable selection of decomposition levels and filters, and (3) incorrect division of validation and calibration data. The readers are encouraged to look up more information about the findings of Quilty and Adamowski (2018). The authors’ concern about the development of MODWT and DWT decomposition were addressed in this study. After separating the DO variables to resolve more comprehensive information to create the MODWT-MARS model, Fig. 3 displays the time-series of the intrinsic mode functions (IMFs) and the residual components and decomposed components of MODWT.

**Fig. 4:**
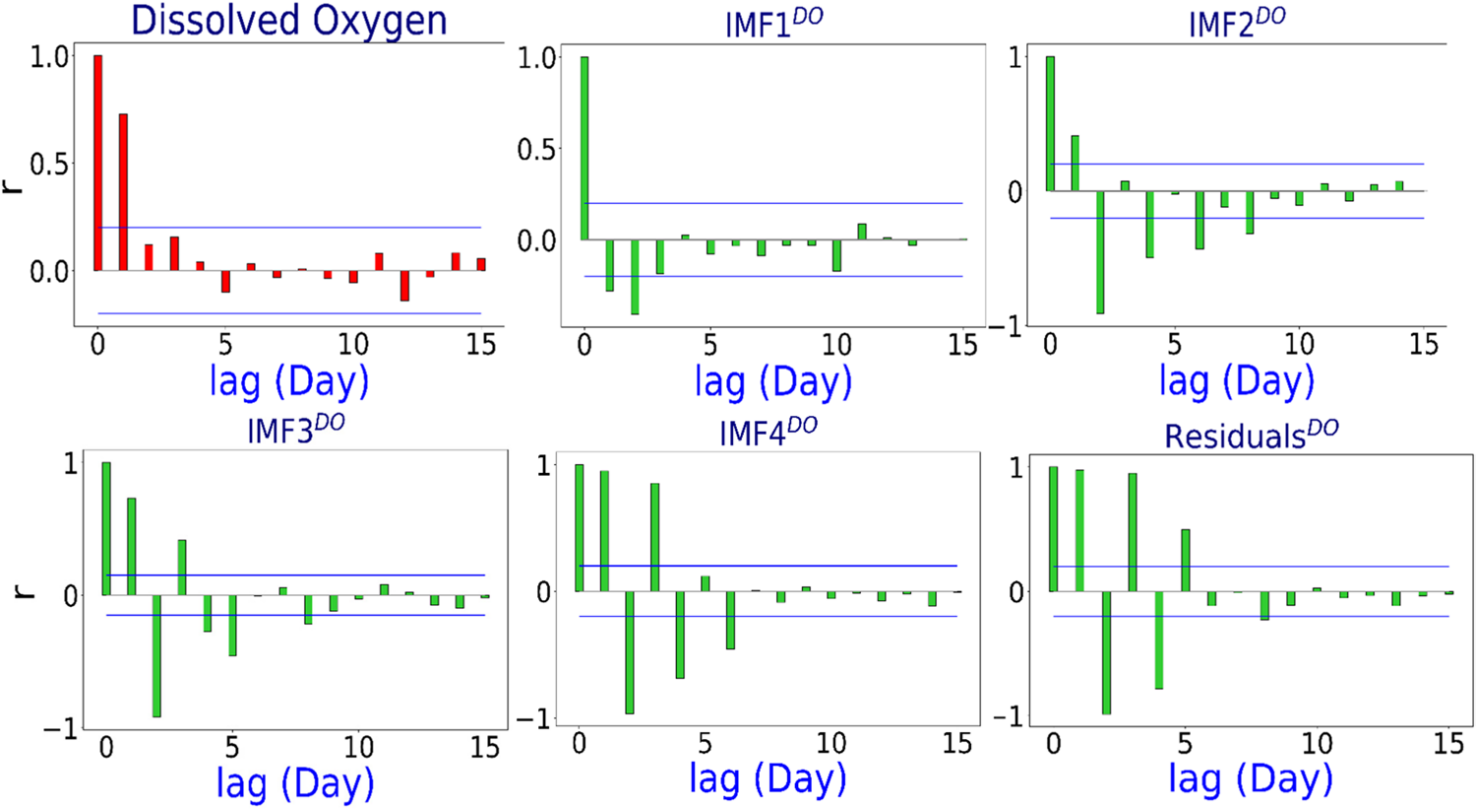
Partial autocorrelation function (PACF) plot of the DO time series exploring the antecedent behaviour in terms of the lag of daily DO. The blue line in the figures indicates the ± 95% confidence level

There is no formula for verifying whether or not a model’s valid predictors are present (Tiwari and Adamowski 2013). Although the research describes three input selection strategies for picking the time series of lagged memories of DO and predictors for an optimum model, the literature does not specify which method should be used. The autocorrelation function (ACF), partial autocorrelation function (PACF), and cross-correlation function (CCF) approaches are the three types of approaches to consider. A substantial antecedent behaviour in terms of the lag of DO from the predictors was found in this study, utilising PACF as the predictor (Tiwari and Adamowski 2013; Tiwari and Chatterjee 2011). Figure 4 demonstrates the PACF for DO time series showing the antecedent behaviour in terms of the lag of DO and decomposed components of DO using MODWT. It is clear from the figure that antecedent monthly delays are found significant.

**Fig. 5:**
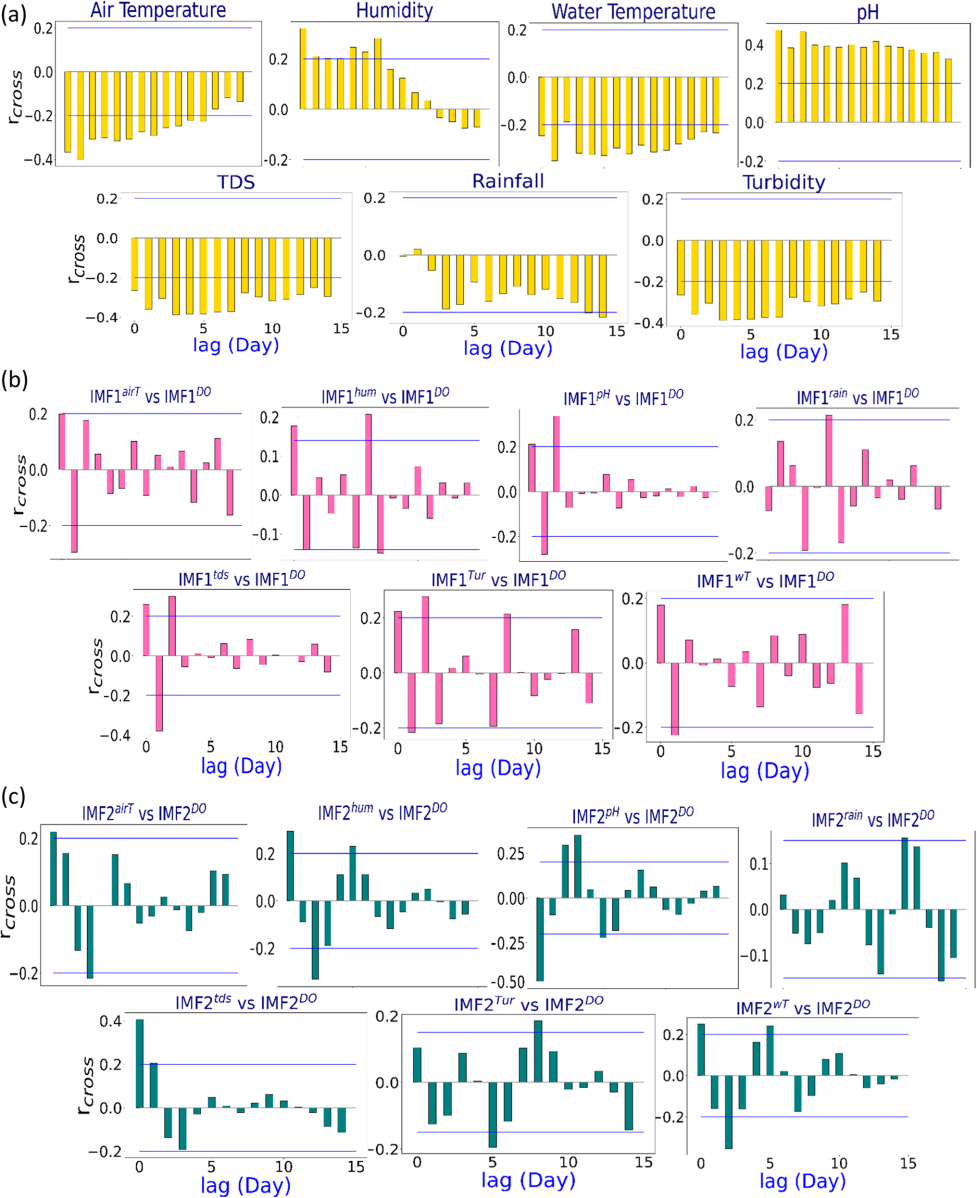

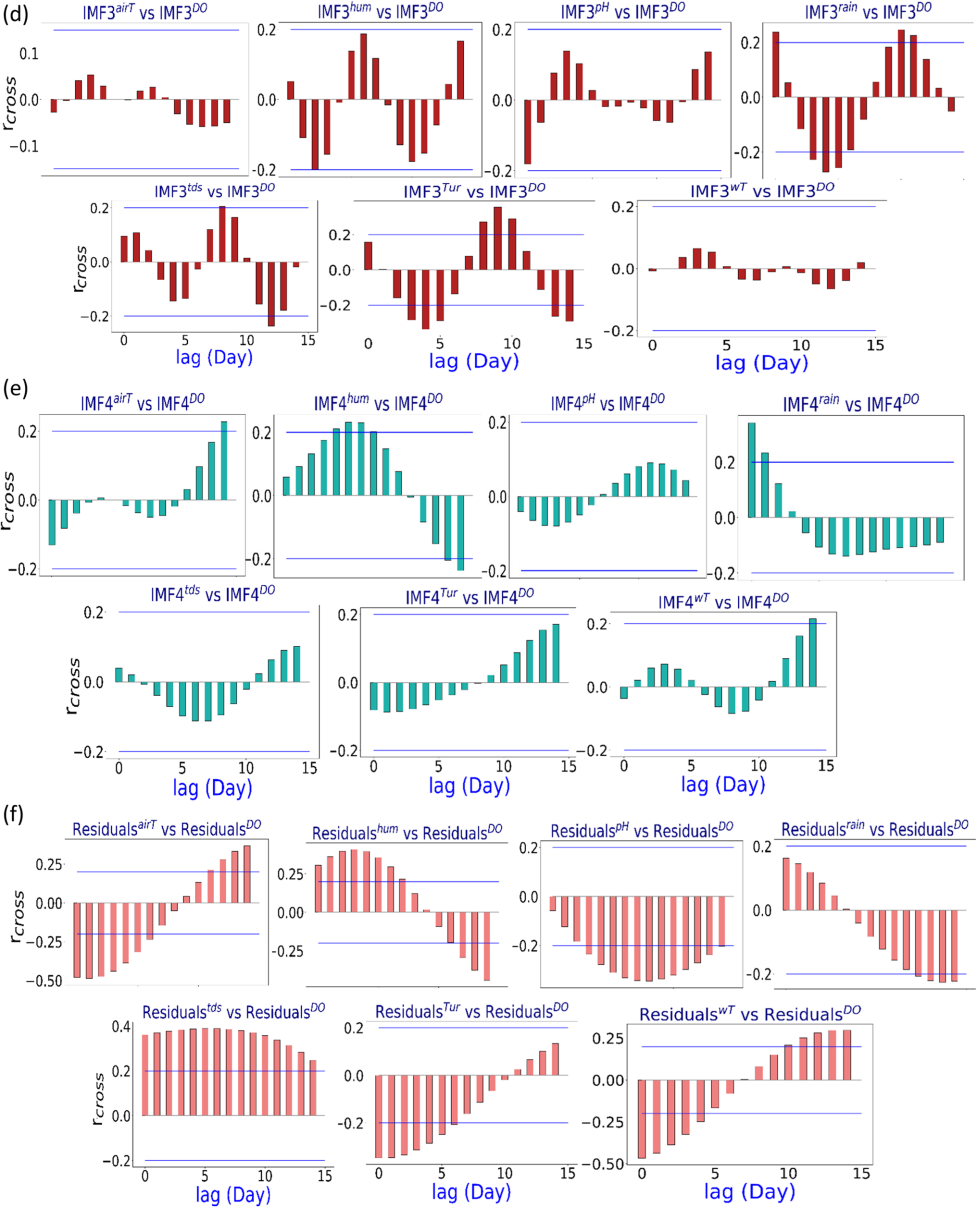
An analysis of the statistically significant cross-correlation function plots of **a** actual variables vs DO, **b** IMF1 of all variables vs IMF1 of DO, **c** IMF2 of all variables vs IMF2 of DO, **d** IMF3 of all variables vs IMF3 of DO, **e** IMF4 of all variables vs IMF4 of DO, f) residuals of all variables vs residuals of DO

The cross-correlation function determines which predictor’s antecedent lag selects the input signal pattern and which pattern the predictor selects (Adamowski et al. 2012). The cross-correlation function is used to establish the statistical similarity between the predictors and the target variable. The cross-correlation function between the predictors and the DO for the River Surma is depicted in Fig. 5a. Afterwards, a set of significant input combinations were determined by assessing *r*_cross_ of each predictor with DO. In this plot, a 95% confidence level of the statistically significant *r*_cross_ is shown in the blue line. It is found from the Fig. 5a the correlation of respective data with DO was found as the highest for all stations at lag zero (*r*_*cross*_* ≈* 0.25–0.45). A similar procedure is maintained for the decomposed predictor variables. Figure 5b–f demonstrate the *r*_cross_ value between *#d*_*1*_ (DO) and *#d*_*n*_ (Predictors) and their respective residuals (*n* = 1 to 4). Figure 5 shows that the r_cross_ value was ranged between 0.25 and 0.50 found more than 95% confidence level. The predictor data sets are normalised (Ahmed 2017; Ali et al. 2019) between 0 and 1 to minimise one variable’s overestimation.

6 $${DO}_{norm}=\frac{DO-{DO}_{min}}{{DO}_{max}-{DO}_{min}}$$

Python-based *Scikit-learn* (Pedregosa et al. 2011a) was used to build this study’s SVR, RF, KRR, BNR, and KNN model. For SVR, the RBF (Radial Basis Function) was employed in developing the SVR model (Suykens et al. 2002). The RBF uses a faster function during training to examine nonlinearities between the objective and predictor variables (Goyal et al. 2014; Lin 2003; Maity et al. 2010). The tricky process of creating an accurate SVR model required identifying the 3D parameters (C, σ, and ε) (Hoang et al. 2014). This is why the NCA algorithm was used to select the parameters with the smallest weight value. (Pedregosa et al. 2011a).

Alternatively, the MARS model adopted the Python-based Py-earth package (Rudy and Cherti 2017). The two MARS models used are cubic or linear piecewise functions. This study used a piecewise cubic model because it provided a smoother response. Also, the generalised recursive partitioning regression was adopted since it can handle multiple preconditioners. A forward and backward selection was used for optimisation. Initially, the algorithm ran with a “naïve” model that only contained the intercept term. The training MSE was reduced by iteratively adding the reflected pairs of basis functions (Table 2).

The accuracy of the hybrid MARS and other comparing models was constructed using piecewise cubic and linear regression functions, respectively. The best MARS model was selected using the lowest Generalised Cross-Validation (GCV) (Lin 2003); the MODWT-MARS model yielded the lowest RMSE and the highest LM, demonstrating the most accurate predictions. The optimum tuning parameters of various machine learning methods are tabulated in Table 3.

**Table 2:** Different input combinations prepared by using the NCA feature selection algorithm. Numerical values after the variable indicate respective lag memories of the datasets

No	Different input combinations
1	h_3_
2	h_3_,tr_5_
3	h_3_,tr_5_, h_5_
4	h_3_,tr_5_, h_5_, h_6_
5	h_3_,tr_5_, h_5_, h_6_, tr_4_
6	h_3_,tr_5_, h_5_, h_6_, tr_4_, tr_7_
7	h_3_,tr_5_, h_5_, h_6_, tr_4_, tr_7_, tr_3_
8	h_3_,tr_5_, h_5_, h_6_, tr_4_, tr_7_, tr_3_, h_4_
9	h_3_,tr_5_, h_5_, h_6_, tr_4_, tr_7_, tr_3_, h_4_, tr_6_
10	h_3_,tr_5_, h_5_, h_6_, tr_4_, tr_7_, tr_3_, h_4_, tr_6_, td_4_
11	h_3_,tr_5_, h_5_, h_6_, tr_4_, tr_7_, tr_3_, h_4_, tr_6_, td_4_, td_2_
12	h_3_,tr_5_, h_5_, h_6_, tr_4_, tr_7_, tr_3_, h_4_, tr_6_, td_4_, td_2_, td_1_
13	h_3_,tr_5_, h_5_, h_6_, tr_4_, tr_7_, tr_3_, h_4_, tr_6_, td_4_, td_2_, td_1_, td_8_
14	h_3_,tr_5_, h_5_, h_6_, tr_4_, tr_7_, tr_3_, h_4_, tr_6_, td_4_, td_2_, td_1_, td_8_, p_4_
15	h_3_,tr_5_, h_5_, h_6_, tr_4_, tr_7_, tr_3_, h_4_, tr_6_, td_4_, td_2_, td_1_, td_8_, p_4_,a_2_
16	h_3_,tr_5_, h_5_, h_6_, tr_4_, tr_7_, tr_3_, h_4_, tr_6_, td_4_, td_2_, td_1_, td_8_, p_4_,a_2_, td_3_
17	h_3_,tr_5_, h_5_, h_6_, tr_4_, tr_7_, tr_3_, h_4_, tr_6_, td_4_, td_2_, td_1_, td_8_,p_4_,a_2_, td_3_, p_3_
18	h_3_,tr_5_, h_5_, h_6_, tr_4_, tr_7_, tr_3_, h_4_, tr_6_, td_4_, td_2_, td_1_, td_8_,p_4_,a_2_, td_3_, p_3_, p_5_
19	h_3_,tr_5_, h_5_, h_6_, tr_4_, tr_7_, tr_3_, h_4_, tr_6_, td_4_, td_2_, td_1_, td_8_,p_4_,a_2_, td_3_, p_3_, p_5_, a_4_
20	h_3_,tr_5_, h_5_, h_6_, tr_4_, tr_7_, tr_3_, h_4_, tr_6_, td_4_, td_2_, td_1_, td_8_,p_4_,a_2_, td_3_, p_3_, p_5_, a_4_, a_1_
21	h_3_,tr_5_, h_5_, h_6_, tr_4_, tr_7_, tr_3_, h_4_, tr_6_, td_4_, td_2_, td_1_, td_8_,p_4_,a_2_, td_3_, p_3_, p_5_, a_4_, a_1_, w_3_
22	h_3_,tr_5_, h_5_, h_6_, tr_4_, tr_7_, tr_3_, h_4_, tr_6_, td_4_, td_2_, td_1_, td_8_,p_4_,a_2_, td_3_, p_3_, p_5_, a_4_, a_1_, w_3_, a_3_
23	h_3_,tr_5_, h_5_, h_6_, tr_4_, tr_7_, tr_3_, h_4_, tr_6_, td_4_, td_2_, td_1_, td_8_,p_4_,a_2_, td_3_, p_3_, p_5_, a_4_, a_1_, w_3_, a_3_, w_2_
24	h_3_,tr_5_, h_5_, h_6_, tr_4_, tr_7_, tr_3_, h_4_, tr_6_, td_4_, td_2_, td_1_, td_8_,p_4_,a_2_, td_3_, p_3_, p_5_, a_4_, a_1_, w_3_, a_3_, w_2_, r_13_
25	h_3_,tr_5_, h_5_, h_6_, tr_4_, tr_7_, tr_3_, h_4_, tr_6_, td_4_, td_2_, td_1_, td_8_,p_4_,a_2_, td_3_, p_3_, p_5_, a_4_, a_1_, w_3_, a_3_, w_2_, r_13_, w_4_
26	h_3_,tr_5_, h_5_, h_6_, tr_4_, tr_7_, tr_3_, h_4_, tr_6_, td_4_, td_2_, td_1_, td_8_,p_4_,a_2_, td_3_, p_3_, p_5_, a_4_, a_1_, w_3_, a_3_, w_2_, r_13_, w_4_, r_1_
27	h_3_,tr_5_, h_5_, h_6_, tr_4_, tr_7_, tr_3_, h_4_,tr_6_,td_4_,td_2_,td_1_,td_8_,p_4_,a_2_, td_3_, p_3_, p_5_, a_4_, a_1_, w_3_, a_3_, w_2_, r_13_, w_4_, r_1_, w_1_
28	h_3_,tr_5_, h_5_,h_6_,tr_4_,tr_7_,tr_3_ h_4_, tr_6_, td_4_, td_2_, td_1_, td_8_,p_4_,a_2_, td_3_, p_3_,p_5_,a_4_,a_1_,w_3_,a_3_,w_2_, r_13_, w_4_, r_1_, w_1_, d_1_
29	h_3_,tr_5_,h_5_,h_6_,tr_4_,tr_7_,tr_3_,h_4_, tr_6_, td_4_, td_2_, td_1_, td_8_,p_4_,a_2_, td_3_, p_3_,p_5_,a_4_,a_1_,w_3_,a_3_, w_2_,r_13_,w_4_,r_1_,w_1_,d_1_,w_5_
30	h_3_, tr_5_, h_5_, h_6_,tr_4_,tr_7_, tr_3_,h_4_,tr_6_,td_4_,td_2_,td_1_,td_8_,p_4_,a_2_,td_3_,p_3_,p_5_,a_4_,a_1_,w_3_,a_3_,w_2_,r_13_,w_4_,r_1_,w_1_,d_1_,w_5_,p_1_

### Model evaluation benchmarks

Several statistical score metrics were considered in the rigorous evaluation of the proposed model (i.e. MODWT-MARS) compared with the counterpart models. The commonly adopted model score metrics such as Pearson’s correlation coefficient (*r*), root-mean square error (RMSE; mg/l), mean absolute error (MAE; mg/l), Nash–Sutcliffe efficiency (NSE), Absolute Percentage Bias (APB; %), and Willmott’s Index agreement (WI) (Krause et al. 2005; Legates and McCabe 1999; Nash and Sutcliffe 1970; Willmott et al. 2012) were used as the popular metrics used elsewhere (Ahmed et al. 2021a; Ghimire et al. 2019c). Due to the stations’ geographic alterations, the percentage error measures relative error values such as RRMSE, RMAE, and MAPE were considered. Owing to the inherent merits and weaknesses of the metrics, combining them is prudent (Sharma et al. 2019). Different sets of model evaluation metrics such as RMSE, MAE, and *r*^2^ (coefficient of determination) (Chu et al. 2020); NSE, RMSE, MAE, and PERS (persistence index) (Tiwari and Chatterjee 2010); Legates-McCabe’s Index (LM), Willmott’s Index (WI), RRMSE, and RMAE (Ali et al. 2019; Ghimire et al. 2019b; Yaseen et al. 2019) were selected for evaluating the model with numerous sets of variables. The correlation coefficient (*r*) provides information about the linear association between forecasted and observed DO data; therefore, it is limited in its capacity. However, *r* is considered oversensitive to extreme values (Willmott et al. 1985). Moreover, RMSE and MAE can provide appropriate information regarding the forecasting skill, whereby RMSE evaluates the robustness of the model related to high values but focuses on the deviation of the forecasted value from the observed (Deo et al. 2017a). Alternatively, MAEs are not a perfect replacement for RMSEs (Chai and Draxler 2014). The Nash–Sutcliffe efficiency (NSE) is a widely used model evaluation criteria for the hydrological models. NSE is a dimensionless metric and a scaled version of MSE, offering a better physical interpretation (Legates and McCabe 2013). However, the NSE over-emphasises the higher values of outliers, and lower values are neglected (Legates and McCabe 1999). Due to the standardisation of the observed and predicted means and variance, the robustness of *r* is limited. Willmott’s Index (WI) was utilised to address this issue by considering the mean squared error ratio instead of the differences. The mathematical notations of the statistical metrics are as follows:

7 $$\mathrm{MAE }(\mathrm{mg}/\mathrm{L})=\frac{1}{N}{\sum}_{i=1}^{N}\left|{DO}_{for}^{i}-{DO}_{obs}^{i}\right|$$

8 $$\mathrm{RMSE }(\mathrm{mg}/\mathrm{L})=\sqrt{\frac{1}{\mathrm{N}}{\sum}_{\mathrm{i}=1}^{\mathrm{N}}{({DO}_{for}^{i}-{DO}_{obs}^{i})}^{2}}$$

9 $$\mathrm{NSE}=1- \left[1- \frac{\sum_{\mathrm{i}=1}^{\mathrm{N}}{{DO}_{for}^{i})}^{2}}{\sum_{\mathrm{i}=1}^{\mathrm{N}}{\left({DO}_{obs}^{i}- {\overline{DO} }_{for}^{i}\right)}^{2}}\right]$$

10 $$\mathrm{r}= {\left\{\frac{\sum_{\mathrm{i}=1}^{\mathrm{N}}\left({DO}_{obs}^{i}-{\overline{DO} }_{obs}^{i}\right)({DO}_{for}^{i}-{\overline{DO} }_{for}^{i})}{\sqrt{{\sum }_{\mathrm{i}=1}^{\mathrm{N}}{\left({DO}_{obs}^{i}-{\overline{DO} }_{obs}^{i}\right)}^{2} {\sum_{\mathrm{i }=1}^{\mathrm{N}}({DO}_{for}^{i}-{\overline{DO} }_{for}^{i})}^{2}}}\right\}}^{2}$$

11 $$\mathrm{RRMSE }(\mathrm{\%})= \frac{\sqrt{\frac{1}{\mathrm{N}}\sum_{\mathrm{i}=1}^{\mathrm{N}}{({DO}_{for}^{i}-{DO}_{obs}^{i})}^{2}}}{\frac{1}{\mathrm{N}}\sum_{\mathrm{i}=1}^{\mathrm{N}}{(DO}_{obs}^{i})} \times 100$$

12 $$\mathrm{RMAE }(\mathrm{\%})= \frac{\frac{1}{\mathrm{N}}\sum_{\mathrm{i}=1}^{\mathrm{N}}\left|{DO}_{for}^{i}-{DO}_{obs}^{i}\right|}{\frac{1}{\mathrm{N}}\sum_{\mathrm{i}=1}^{\mathrm{N}}{DO}_{obs}^{i})} \times 100$$

13 $$\mathrm{MAPE }(\mathrm{\%})=\frac{1}{\mathrm{N}} \left({\sum}_{\mathrm{i}=1}^{\mathrm{N}}\left|\frac{{DO}_{for}^{i}-{DO}_{obs}^{i}}{{DO}_{obs}^{i}} \right|\right)*100$$

14 $$\mathrm{WI }= 1- \left[\frac{\sum_{\mathrm{i}=1}^{\mathrm{N}}{({DO}_{for}^{i}-{DO}_{obs}^{i})}^{2}}{\sum_{\mathrm{i}=1}^{\mathrm{N}}{\left(\left|{DO}_{for}^{i}-{\overline{DO} }_{obs}^{i}\right|+ \left|{DO}_{obs}^{i}-{\overline{DO} }_{obs}^{i}\right| \right)}^{2}}\right]$$

15 $$\mathrm{APB }(\mathrm{mg}/\mathrm{l}) = \left[\frac{\sum_{\mathrm{i}=1}^{\mathrm{N}}\left(\left|{DO}_{for}^{i}-{DO}_{obs}^{i}\right|\right)*100}{\sum_{\mathrm{i}=1}^{\mathrm{N}}\left|{DO}_{obs}^{i}\right|}\right]$$

16 $$\mathrm{LM} = 1- \left[\frac{\sum_{\mathrm{i}=1}^{\mathrm{N}}\left|{DO}_{for}^{i}-{DO}_{obs}^{i}\right|}{\sum_{\mathrm{i}=1}^{\mathrm{N}}\left|{DO}_{obs}^{i}-{\overline{DO} }_{obs}^{i}\right|}\right]$$

where ${DO}_{obs}^{i}$ and ${DO}_{for}^{i}$ denote the observed and model-forecasted values from the *i*th element; ${\overline{DO} }_{obs}^{i}$ and ${\overline{DO} }_{for}^{i}$ denote their average, respectively, and N represents the observation’s number of the DO.

## Results

In this study, MARS models optimised using a feature decomposition approach were utilised to forecast DO time series using hydro-meteorological variables. Several ways were employed to do this, including the conventional machine learning models (i.e. MARS, RF, SVR, KNN, and KRR), feature decomposition methods (i.e. MODWT, CEEMDAN, EEMD, EMD, and DWT), and the feature selection method (i.e. NCA) to screen the optimal model to forecast the DO. Though the mathematical metrics are so ambiguous that there is no way to evaluate the suitable alternative, it is reasonable to use multiple performance evaluation approaches. Compared to the other models, the hybrid, and standalone models of BNR, KNN, KRR, and RF outstripped all decomposition methods. The performance of MODWT-MARS has revealed that the NCA algorithm helped choose the relevant features to assist the MARS in better emulating the future DO concentration. MODWT found important performance matrices, such as r, NSE, WI, RMSE, and MAE. The MODWT-MARS model outperforms all the other tested models.

This study used the NCA algorithm to screen the appropriate predictor variables in the model. Table 2 provides the input combination for forecasting DO. The robustness of the NCA integrated BNR, KNN, KRR, MARS, and SVR model is provided in Tables A1–A6 in terms of statistical metrics would be found as supplementary materials. Tables show that each model’s optimum standalone models were found between combinations from 19 to 29. For the case of the BNR model, the standalone model (BNR_28_) shows poor performance (*r* = 0.809, WI = 0.887, RMAE = 7.55%, and MAE = 0.275) comparing with the BNR-MODWT model (*r* = 0.977, WI = 0.987, RMAE = 3.37%, and MAE = 0.117). Moreover, the hybrid models showed improved performance ranging from 0.888 to 0.977 and 7.17 to 3.37% for *r* and RMAE accordingly. The MARS_29_ model was found as the optimum model (*r* = 0.824, WI = 0.895, RMAE = 7.97%, and MAE = 0.277) among all combinations of MARS model. The MODWT-MARS model was found as the highest performed model with substantial performance parameters (*r* = 0.981, WI = 0.990, RMAE = 2.47%, and MAE = 0.089) which is followed by CEEMDAN-MARS model (*r* = 0.949, WI = 0.971, RMAE = 4.65%, and MAE = 0.156). Mentionable that the highest model of SVR was found for CEEMDAN-SVR (*r* = 0.971, WI = 0.983, RMAE = 3.36%) compared to the optimum standalone model (SVR_20_). Mentionable that KRR, KNN, and RF model provides poor performance comparatively.

**Table 3:** The optimal hyperparameter of the proposed MARS model, including that of the other benchmark models’ methods include machine learning (i.e. BNR, SVR, KRR, KNN, and RF)

Name	Hyper-parameters	Acronym	Optimum
KRR	Regularisation strength	*alpha*	*1.5*
	Kernel mapping	*kernel*	*linear*
	Gamma parameter	*gamma*	*None*
	Degree of the polynomial kernel	*degree*	*3*
	Zero coefficient for polynomial and sigmoid kernels	*coef0*	*1.2*
BNR	Maximum number of iterations	*n_iter*	*200*
	Stop the algorithm if w has converged	*tol*	*0.0001*
	Shape parameter for Gamma distribution over alpha	*alpha_1*	*1e-05*
	Inverse scale parameter over alpha	*alpha_2*	*1e-05*
	Shape parameter for Gamma distribution over lambda	*lambda_1*	*1e-06*
	Inverse scale parameter for Gamma distribution over lambda	*lambda_2*	*1e-04*
	The initial value for alpha	*alpha_init*	*None*
KNN	Number of neighbours	*n_neighbors*	*5*
	Weights	*Weights*	*uniform*
	The algorithm used to compute the nearest neighbours	*algorithm*	*auto*
	Leaf-size passed	*leaf_size*	*30*
	Power parameter for the Minkowski metric	*p*	*2*
	The distance metric to use for the tree	*metric*	*minkowski*
	Additional keyword arguments for the metric	*metric_params*	*none*
	The number of parallel jobs	*n_jobs*	*int*
MARS	maximum degree of terms	*max_degree*	*1*
	Smoothing parameter used to calculate GCV	*penalty*	*3.0*
RF	Number of trees in the forest	*n_estimators*	*120*
	Maximum depth of the tree	*max_depth*	*2*
	Minimum number of samples for an internal node	*min_sample_split*	*2*
	Number of features for the best split	*max_features*	*auto*

**Fig. 6:**
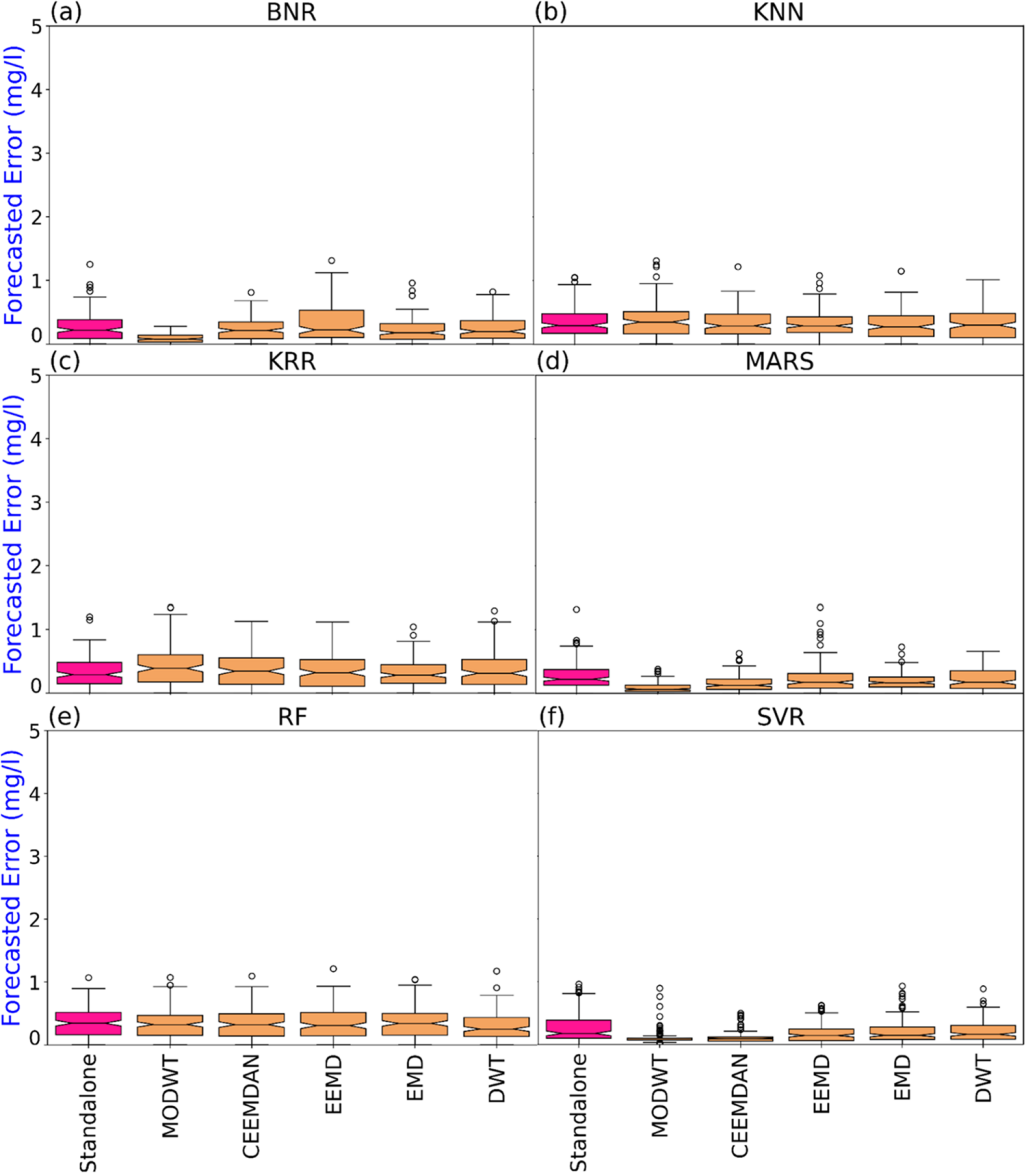
Box plots of hybrid models (MODWT-MARS) and their respective standalone counterparts (i.e. MARS, BNR, KRR, KNN, RNN, and SVR) in forecasting DO compare to the observed DO of Surma River

Further analysis through a box plot showing the forecasted vs observed DO and absolute forecasting error of all hybrid models is illustrated in Fig. 6. The absolute forecasted error was determined as |FE|= DO^for^ – DO^obs^. The box plot demonstrates the observed (DO^obs^) data dispersion and forecasted (DO^for^) DO from the proposed machine learning approaches and comparing models. Figure 6b, c, and e visualise the quartiles’ data with distinctly larger outliers. The lower end of the plot lies between the lower quartile (25^th^ percentile) and the upper quartile (75th percentile). The MODWT-MARS model shows an identical prediction compared with MODWT-SVR, with higher outliers for the SVR model. A more in-depth inspection of the absolute forecast error (|FE|) from the hybrid MODWT-MARS model further strengthens the suitability of the hybrid MARS approach in predicting the DO of the Surma River, which has the narrowest distribution compared with other models. The MODWT-MARS model has a significant percentage (98%) of the |FE| in the first error brackets (0 <|FE|< 0.25), while the MODWT-SVR model has a percentage of 95%.

**Fig. 7:**
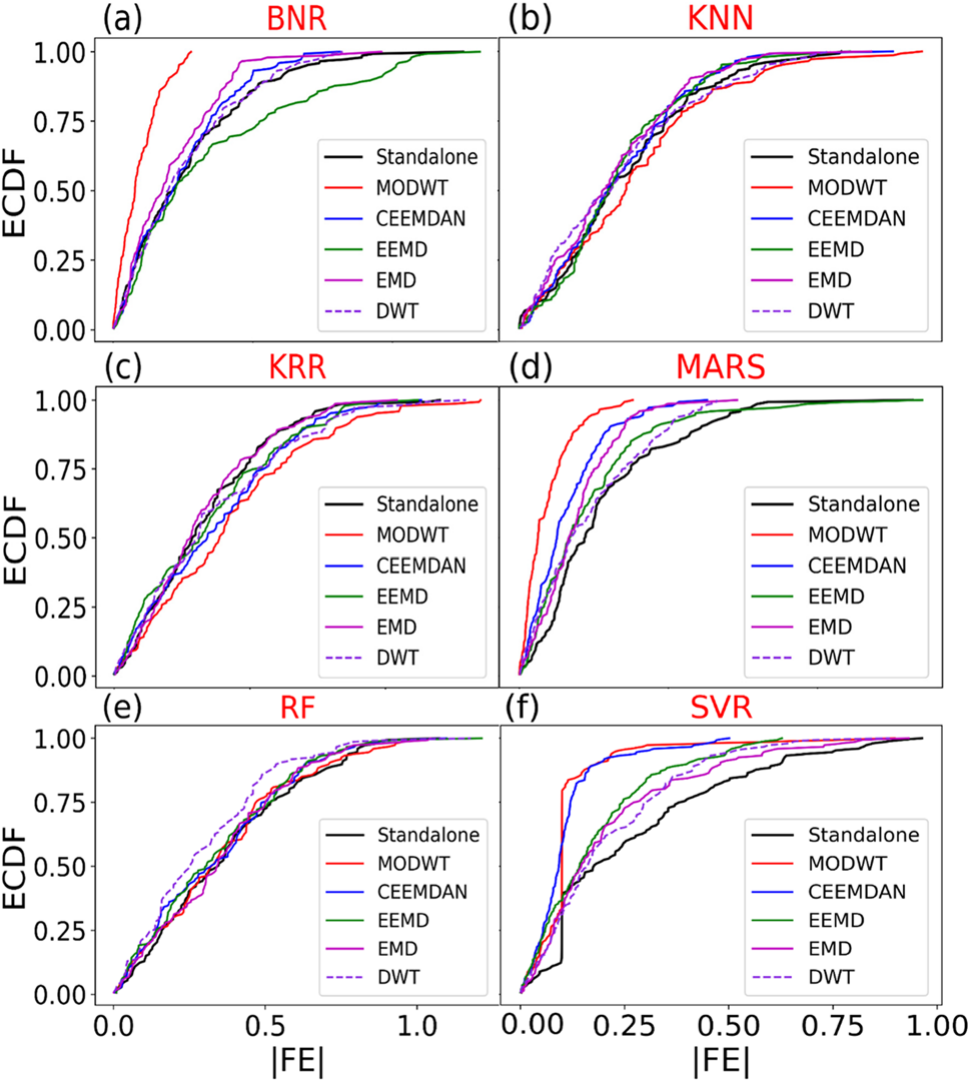
Empirical cumulative distribution function (CDF) of forecasted error |FE| of DO generated by the proposed MODWT-MARS and comparing models

The empirical cumulative distribution function (ECDF) visualisation demonstrates the forecast error data’s feature from the least to highest and perceives the full features circulated across the dataset. Figure 7 represents the empirical CDF of all six models for objective models and comparing models. The hybrid MODWT-MARS model was seen as reasonably sound against other models. The MODWT-MARS generated errors significantly lower from 0 to 0.25 mg/l. In the model-like KNN, KRR, and RF, the distribution of CDF was larger comparatively. The analysis also revealed that the standalone models showed a poor distribution, proving that MODWT-MARS was the most precise and responsive model.

**Fig. 8:**
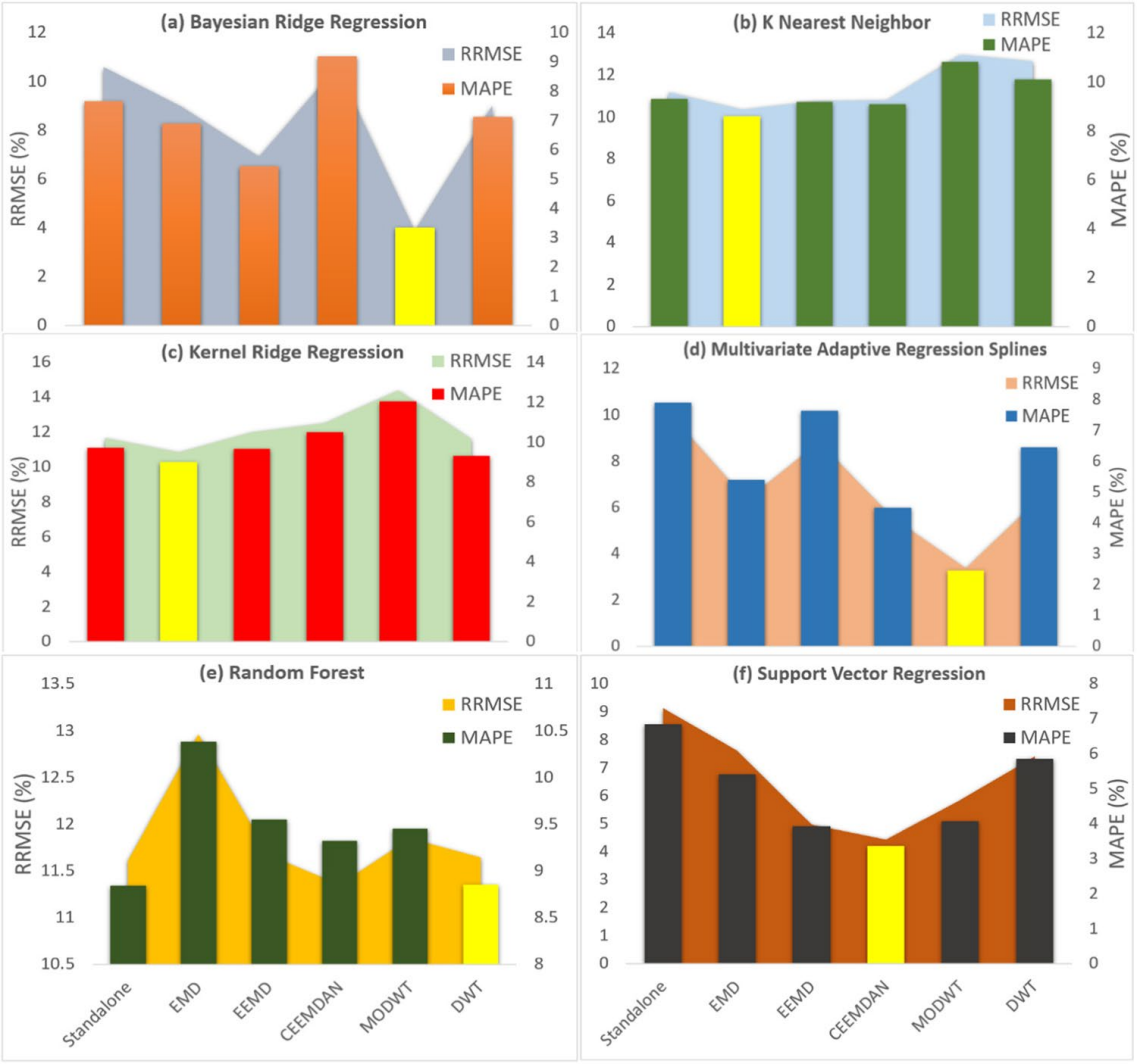
Comparison of the forecasting skill of proposed models in terms of RRMSE (%) and MAPE (%) in the testing period

To analyse the proposed MODWT-MARS model’s further robustness, the models’ forecasting performance was further assessed based on RRMSE and MAPE for all tested models, as shown in Fig. 8. From the figure, the magnitude of RRMSE and MAPE for the objective model (MODWT-MARS) is significantly lower, which clarifies the potential merits of the proposed model. The best RRMSE (3.6%) and MAPE (2.2%) were found for the MODWT-MARS model, which was followed by the MODWT-BNR model with moderate RRMSE (4.0%) and MAPE (3%). Besides, KNN, KRR, and RF models with MODWT showed RRMSE (11.5% to 13.5%) and MAPE (9.5% to 12%) values, demonstrating poor performance.

**Fig. 9:**
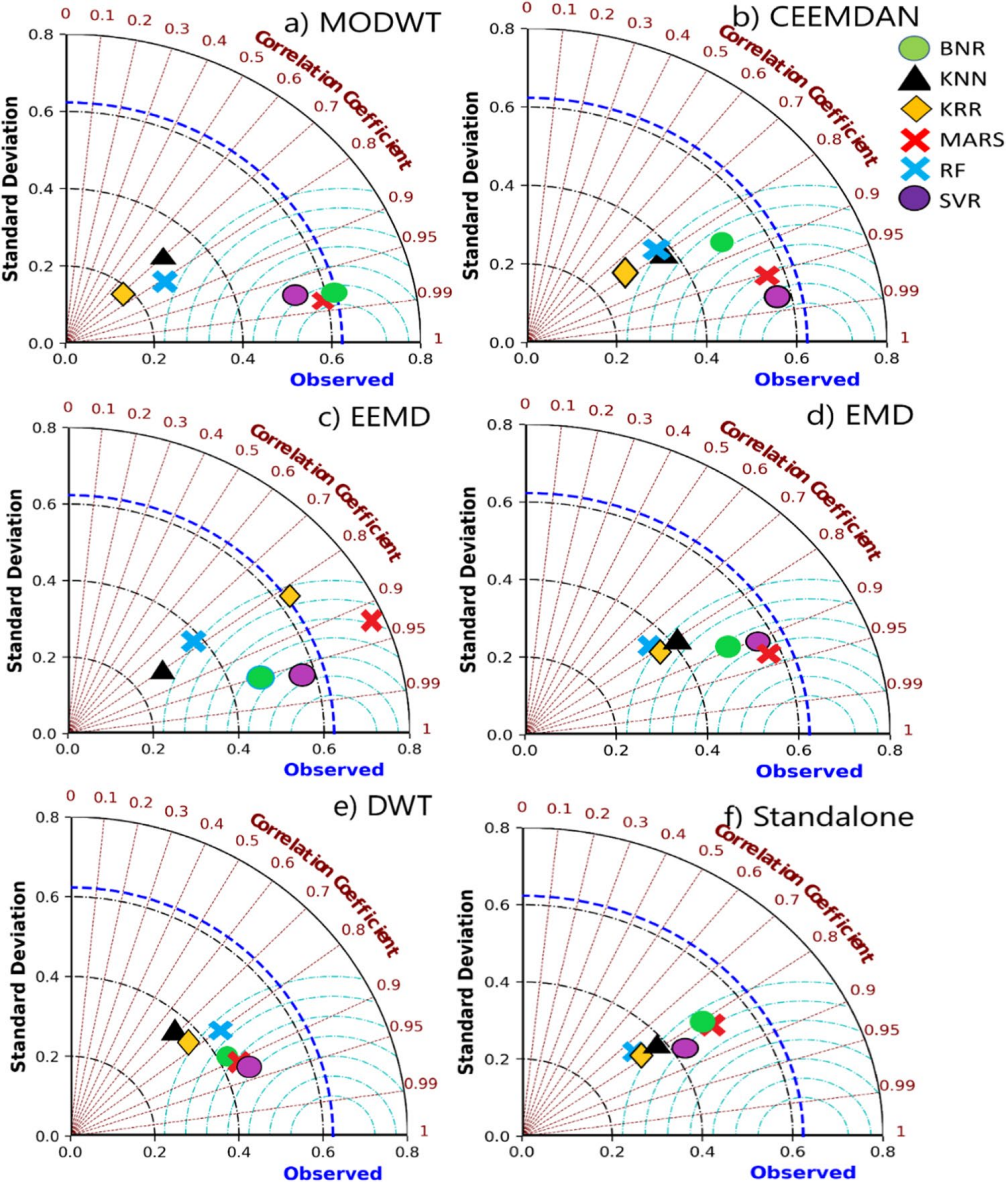
Tylor diagram representing correlation coefficient and the standard deviation difference for proposed hybrid models vs benchmark models

Compared to the standalone models using the Taylor diagram (Taylor 2001), the proposed model performance improves the interpretation presented in Fig. 9. The Taylor diagram demonstrates that the MODWT-MARS model with the NCA algorithm is closer to the observation than the comparing models. Again, the forecasted DO illuminates the proposed model’s better pertinency than the standalone and benchmark models. The benchmark models’ performance with CEEMDAN and MODWT (i.e. MODWT-SVR, CEEMDAN-SVR, and CEEMDAN-MARS) achieved closer proximity to the observed values. However, the proximity of the observed DO for the MARS model with MODWT feature decomposition is the closest.

**Fig. 10:**
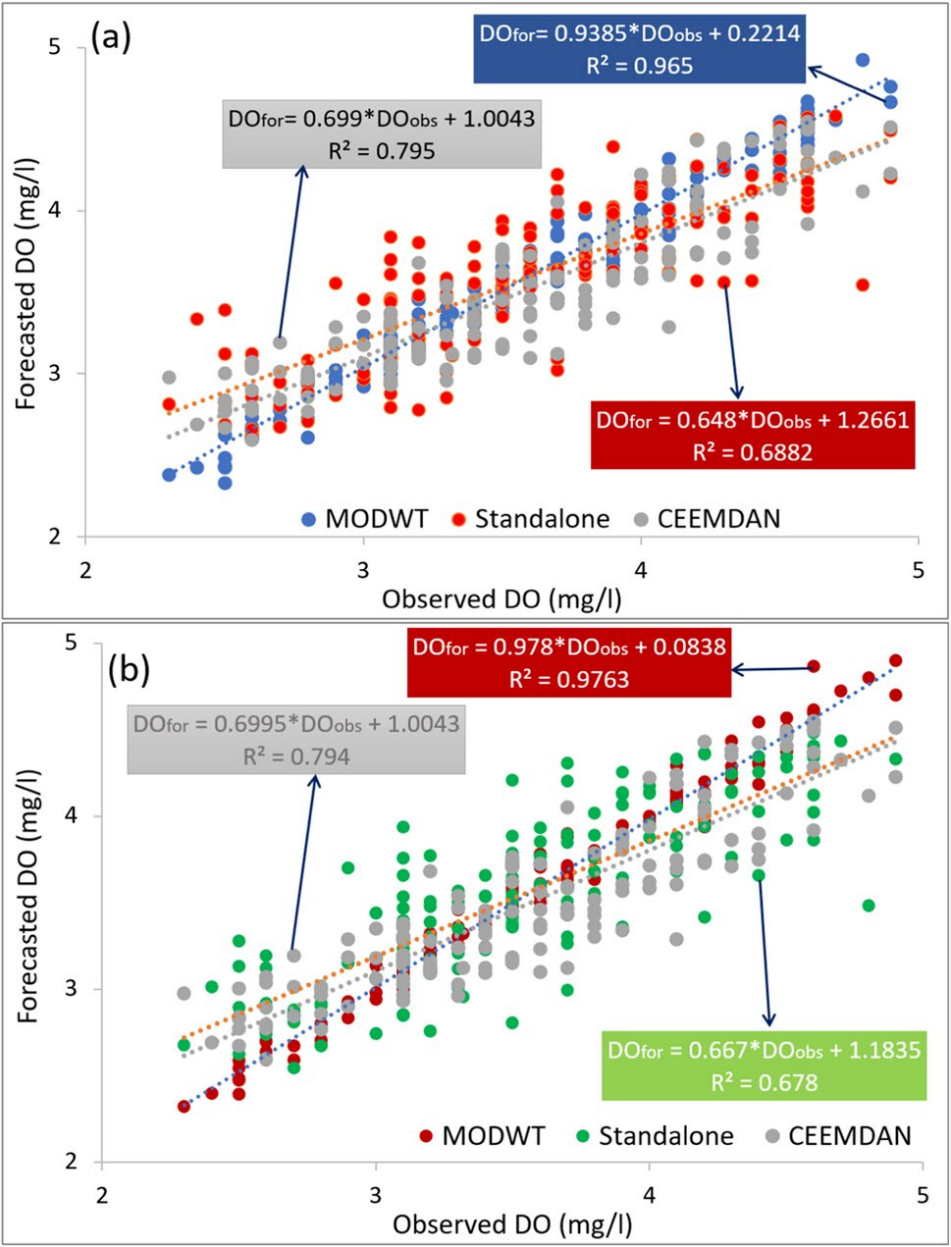
Scatter plot of forecasted vs observed DO, using **a** Bayesian ridge regression (BNR) and **b** multiple adaptive regression splines model using MODWT and CEEMDAN decomposition. A least square regression line and coefficient of determination (*R*^2^) with a linear fit equation are shown in each sub-panel

The scatter plot of the forecasted and observed DO for the proposed MODWT-MARS model portrayed a detailed comparison of DO forecasting (Fig. 10). The scatter plots comprise with the coefficient of determination (R^2^) with goodness-of-fit between forecasted vs observed DO and a least-square fitting line and the corresponding equation*; DO*_*for*_ = *m X DO*_*obs*_ + *C*, where, *m* is referred to as the gradient, and *C* is denoted as the y-intercept. Figure 10 reveals that the proposed model displays significant performance with a more considerable *R*^2^ value. The DO forecasting using a hybrid machine learning model *(*i.e. MODWT-MARS) performed significantly better than the other models. The magnitudes registered from the hybrid MODWT-MARS model were the closest to unity, which, in pairs (*m|R*^2^), are 0.978|0.976, followed by MODWT-SVR (0.939|0.965). Moreover, the CEEMDAN-SVR (0.699|0.795) and CEEMDAN-MARS (0.700|0.794) models provide a comparatively lower pair. Alternatively, y-intercepts [*ideal value* = *0*] was found close to zero i.e. 0.084 for the proposed model. However, the y-intercept deviated from the ideal value with more outliers for the other models.

**Fig. 11:**
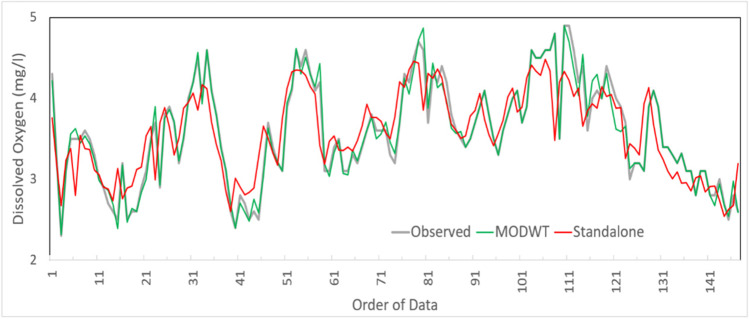
Comparison between forecasted DO and observed DO during model testing using MODWT-MARS and Standalone MARS model

To attain a different interpretation of the proposed MODWT-MARS model’s accuracy, the time series plot is used to comprehend the proposed model’s forecasting ability. Figure 11 demonstrates the time series plot of forecasted and observed DO with MODWT-MARS compared to the standalone MARS model. Results show that the proposed MODWT-MARS model is found close to the observed DO revealed a high predictive accuracy. After applying the NCA algorithm as a feature selection approach and MODWT as a feature decomposition technique, the forecasted DO is enhanced.

**Fig. 12:**
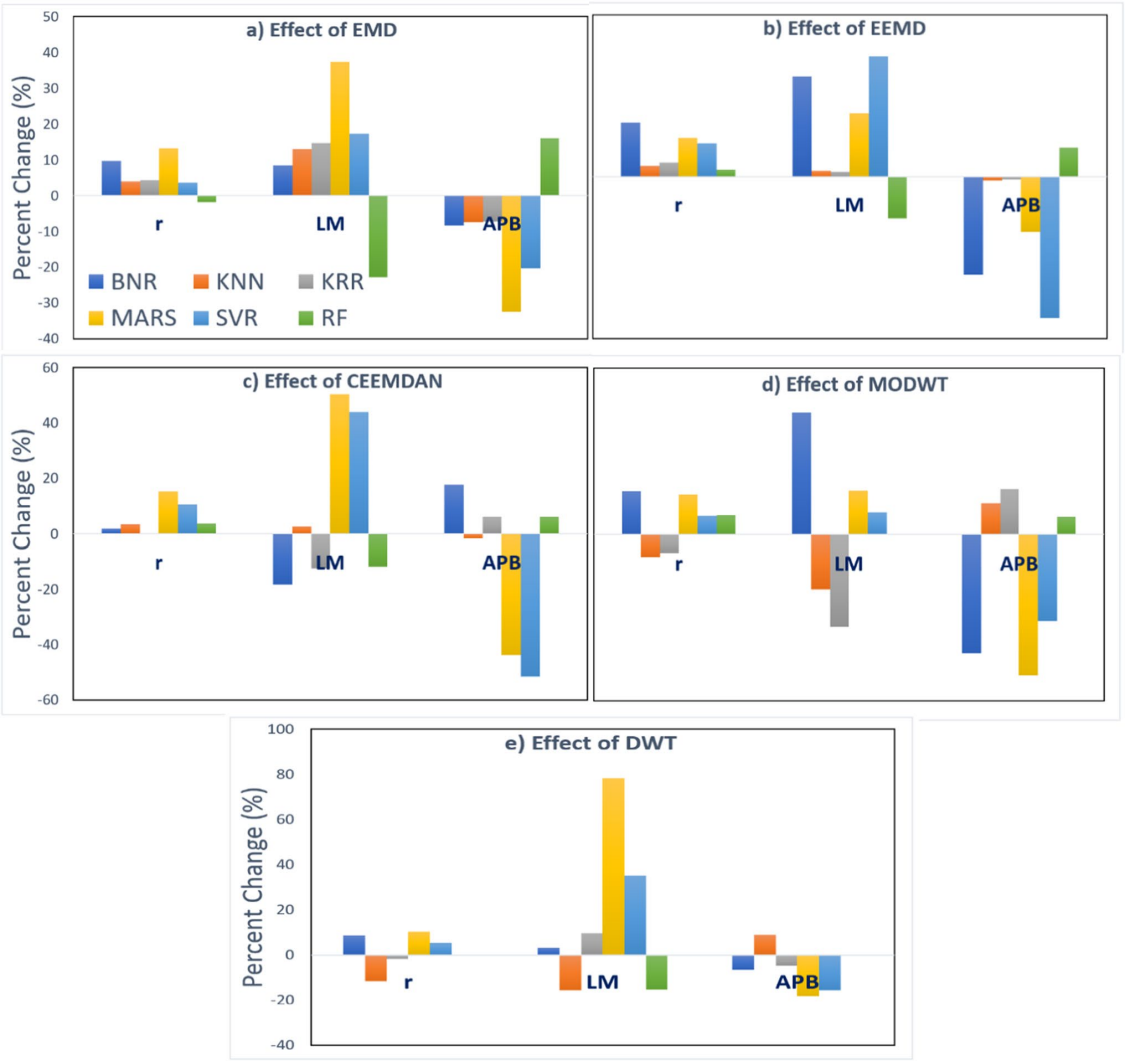
Effect of **a** EMD, **b** EEMD, **c** CEEMDAN, **d** MODWT, and **e** DWT of the performance of six models based on *r*, LM, and APB

Notably, five unique decomposition algorithms, EMD, EEMD, CEEMDAN, DWT, and MODWT, are incorporated to enhance the MARS-based predictive model. In terms of *r*, LM, and APB of DO forecasting, the MODWT effectively forecasts improvement (Fig. 12). In the MARS model, *r* and LM values using the MODWT model increased by ~ 19% and ~ 20% accordingly, and APB decreased by ~ 68%. Similarly, for the BNR model, MODWT feature decomposition skill increased *r* and LM values up to ~ 21% and ~ 59% accordingly, and APB is decreased by ~ 57%. Additionally, *r* and LM values for the MARS model with CEEMDAN are increased by ~ 15% and ~ 50%, respectively. Similarly, the inclusion of DWT, EMD, and EEMD also substantially improved the *r*, LM, and APB values.

## Discussion

According to the findings of this study, different input combinations have varying effects on the outcomes. Then, several input variables must be analysed, and the most appropriate collection of variables must be employed to optimise the products. Every model should have its ideal combination; yet the most effective combination is rare throughout the various models. Al-Musaylh et al. (2019) used the hybrid MARS model in forecasting electricity demand with a good performance. This study demonstrated profound forecasting of Dissolved Oxygen (DO) concentration. Our findings have led to better forecasting than any algorithm evaluated in standalone and hybrid versions. We propose more studies to forecast DO using wet and dry season datasets and compare the results with the whole dataset’s findings. Different pre-processing techniques could also enhance the projection accuracy of the MARS model. First, it is possible to implement a suitable feature selection approach such as NCA (Ahmed et al. 2021a; Ghimire et al. 2019a) algorithm to pick the input variables that significantly impact the model. The feature weight calculated using neighbourhood component analysis (NCA) respective to predictor variables was added one by one based on the highest to lowest feature weight to improve the model performance. The optimum combination of input parameters was found significantly in the proposed hybrid MARS model. By fitting piecewise linear regressions, MARS essentially creates flexible models by approximating the nonlinearity of a model using discrete linear regression slopes in various intervals of the independent variable space. An expansion in product spline basis functions of the predictors selected during a forwards and backwards recursive partitioning technique is how MARS best fits a model given a collection of predictor variables.

The time complexity of machine learning models is very important for the better application of the ML models. All the ML models used in our study shows less complex in terms of training time with less than 2 min for almost all the models. The incorporation of five feature decomposition approaches is vital to understanding the diverse implementation of the models in association with data pre-processing (i.e. feature selection and feature decomposition). The results showed that the inclusion of feature decomposition methods such as MODWT, CEEMDAN, EEMD, EMD, and DWT increased the performance of DO forecasting compared to the respective standalone methods. As MODWT can handle any sample size, the smooth and detail coefficients of MODWT filters and produces a more asymptotically efficient wavelet variance estimator than the DWT. However, the MODWT-MARS model was found as the optimum. Different researchers reported similar performance, where MODWT data decomposition is reported to improve performance (Li et al. 2017a; Prasad et al. 2017).

## Conclusions

This study developed hybrid machine learning models incorporating neighbourhood component analysis (NCA) as a feature selection method, multivariate adaptive regression splines (MARS) as a predictive model, and MODWT as a feature decomposition method for DO forecast of the river Surma. The study used five distinct feature decomposition approaches (i.e. MODWT, CEEMDAN, EEMD, EMD, and DWT) and six machine learning models (i.e. BNR, KNN, KRR, MARS, RF, and SVR) for developing the optimum model. A new approach to the DO forecasting model was created using a decedent-lagged memory framework to explain the forecasting problem more appropriately and its consequences afterwards. The proposed MODWT-MARS approach provides the optimal performance among the benchmarked models. From this analysis, the following observations can be made.

1. The achieved results demonstrated that the NCA algorithm would be a helpful option for getting the predictor variables’ substantial features. The model’s performance metrics indicate that the NCA algorithm was a suitable tool for feature selection, as the NCA and MODWT optimised models showed better performance than the standalone models.
2. The proposed hybrid MODWT-MARS model outperformed all other models in forecasting the dissolved oxygen concentration of the Surma River. A low MAE (0.089) and a high NS (0.990) value substantiate the MODWT-MARS model’s superiority. Correlation coefficient (r) values increased by 20%, and LM index values increased by 19% compared to their respective standalone models. To be more precise, the MODWT-MARS performed the best when r (0.981), WI (0.990), RMSE (0.121 mg/l), and MAE (0.089 mg/l) values were considered.
3. Based on the analysis, it is recognised that the hybrid MODWT-MARS model with the NCA feature algorithm shows superior forecasting of DO. The station’s antecedent values of water quality parameters and hydro-meteorological variables embed the machine learning approach’s future forecasting success. So, this type of forecast is used for better water quality management.
4. The current analysis strongly implies that MODWT and NCA methods with the MARS model can be used to forecast accurately. Their employment improved the accuracy of the MODWT-MARS model established in the current study and reduced the model’s complexity by removing unnecessary input variables by incorporating NCA.

In addition to providing scientific benefits, the MARS low input need combined with their substantial predictive capability also provided significant practical benefits. They enable the development of a station-specific prudent predictive model of DO for monitoring river health at a minimal cost and the development of region-specific management plans across a range of land use and land cover gradients in a cost-effective manner.

## Supplementary Information

Below is the link to the electronic supplementary material.

Supplementary file1 (DOCX 49 KB)

## Data Availability

This article presents an original research work executed by the authors, so all the data presented depend on their findings and analysis techniques. The datasets used in this article are available from the corresponding author on reasonable request.

## Nomenclature

ACF: Autocorrelation function
ANN: Artificial neural network
BF: Basis functions
BNR: Bayesian ridge regression
BOD: Biological oxygen demand
COD: Chemical oxygen demand
CCF: Cross-correlation function
CEEMDAN: Complete ensemble empirical mode decomposition with adaptive noise
CEEMDAN-BNR: Hybrid model integrating the CEEMDAN algorithm with BNR
CEEMDAN-KNN: Hybrid model integrating the CEEMDAN algorithm with KNN
CEEMDAN-KRR: Hybrid model integrating the CEEMDAN algorithm with KRR
CEEMDAN-MARS: Hybrid model integrating the CEEMDAN algorithm with MARS
CEEMDAN-RF: Hybrid model integrating the CEEMDAN algorithm with RF
CEEMDAN-SVR: Hybrid model integrating the CEEMDAN algorithm with SVR
EEMD: Ensemble empirical mode decomposition
EEMD-BNR: Hybrid model integrating the EEMD algorithm with BNR
EEMD-KNN: Hybrid model integrating the EEMD algorithm with KNN
EEMD-KRR: Hybrid Model integrating the EEMD algorithm with KRR
EEMD-MARS: Hybrid Model integrating the EEMD algorithm with MARS
EEMD-RF: Hybrid model integrating the EEMD algorithm with RF
EEMD-SVR: Hybrid model integrating the EEMD algorithm with SVR
EMD: Empirical mode Decomposition
EMD-BNR: Hybrid model integrating the EMD algorithm with BNR
EMD-KNN: Hybrid model integrating the EMD algorithm with KNN
EMD-KRR: Hybrid model integrating the EMD algorithm with KRR
EMD-MARS: Hybrid model integrating the EMD algorithm with MARS
EMD-RF: Hybrid model integrating the EMD algorithm with RF
EMD-SVR: Hybrid model integrating the EMD algorithm with SVR
DWT: Discrete wavelet Transformation
DWT-BNR: Hybrid model integrating the DWT algorithm with BNR
DWT-KNN: Hybrid model integrating the DWT algorithm with KNN
DWT-KRR: Hybrid model integrating the DWT algorithm with KRR
DWT-MARS: Hybrid model integrating the DWT algorithm with MARS
DWT-RF: Hybrid model integrating the DWT algorithm with RF
DWT-SVR: Hybrid model integrating the DWT algorithm with SVR
ECDF: Empirical cumulative distribution function
ELM: Extreme learning machine
FE: Forecasting error
GCV: Generalised cross-validation
IMF: Intrinsic mode functions
KNN: K-nearest neighbourhood
KRR: Kernel ridge regression
LM: Legates-McCabe’s Index
LSSVM: Least square support vector machine
MAE: Mean absolute error
MAPE: Mean absolute percentage error
MARS: Multivariate adaptive regression splines
MLP: Multi-layer perceptron
MODWT: Maximum overlap discrete wavelet transformation
MODWT -BNR: Hybrid Model integrating the MODWT algorithm with BNR
MODWT -KNN: Hybrid Model integrating the MODWT algorithm with KNN
MODWT -KRR: Hybrid Model integrating the MODDWT algorithm with KRR
MODWT –MARS: Hybrid Model integrating the MODWT algorithm with MARS
MODWT -RF: Hybrid Model integrating the MODWT algorithm with RF
MODWT -SVR: Hybrid Model integrating the MODWT algorithm with SVR
MRA: Multi-resolution analysis
MSE: Mean squared error
NCA: Neighbourhood component analysis
NSE: Nash–Sutcliffe efficiency
PACF: Partial auto–correlation function
r: Correlation coefficient
RBF: Radial basis function
RF: Random forest
RMSE: Root-mean-square-error
RRMSE: Relative root-mean-square error
SVR: Support vector regression
TDS: Total dissolved solids
WQ: Water quality
