# Supplementary material for: The development of dissolved oxygen forecast model using hybrid machine learning algorithm with hydro-meteorological variables
Source: Environ Sci Pollut Res Int. 2022 Sep 1;30(3):7851–73. doi: 10.1007/s11356-022-22601-z (PMC9894995; doi:10.1007/s11356-022-22601-z)
Supplement: Supplementary file 1 — Supplementary file1 (DOCX 49 KB) [file 11356_2022_22601_MOESM1_ESM.docx]

**Table 1** Evaluation of the performance of hybrid BNR models *vs* the MARS models with the correlation coefficient (*r*), root mean square error (*RMSE*), mean absolute error (*MAE*), relative mean absolute error (R*MAE*) & Willmot’s Index (*WI*) between the forecasted and observed *DO* in the testing phase. Blue coloured raw indicates the optimum model for the standalone model, and red coloured indicates the best-performing hybrid model.

| **Model** | **r** | **WI** | **RMSE mg/l)** | **MAE (mg/l)** | **RMAE (%)** | **Performance** |
| --- | --- | --- | --- | --- | --- | --- |
| BNR_4_ | 0.520 | 0.605 | 0.561 | 0.445 | 12.505 | ↑ |
| BNR_6_ | 0.536 | 0.626 | 0.554 | 0.448 | 12.467 | ↑ |
| BNR_8_ | 0.519 | 0.641 | 0.557 | 0.442 | 12.646 | ↓ |
| BNR_15_ | 0.698 | 0.788 | 0.476 | 0.373 | 10.117 | ↑ |
| BNR_18_ | 0.705 | 0.795 | 0.472 | 0.366 | 9.928 | ↑ |
| BNR_22_ | 0.724 | 0.811 | 0.457 | 0.352 | 9.563 | ↑ |
| BNR_25_ | 0.729 | 0.814 | 0.454 | 0.353 | 9.615 | ↑ |
| BNR_26_ | 0.722 | 0.811 | 0.456 | 0.354 | 9.627 | ↓ |
| **BNR_28_** | **0.809** | **0.887** | **0.386** | **0.275** | **7.551** | ↑ |
| KNN-EMD | 0.888 | 0.936 | 0.326 | 0.253 | 7.168 | ↑ |
| KNN-EEMD | 0.945 | 0.957 | 0.251 | 0.192 | 5.118 | ↑ |
| KNN-CEEMDAN | 0.829 | 0.879 | 0.385 | 0.325 | 9.043 | ↓ |
| KNN-DWT | 0.878 | 0.904 | 0.322 | 0.253 | 7.339 | ↓ |
| **KNN-MODWT** | **0.977** | **0.987** | **0.140** | **0.117** | **3.370** | ↑ |

**Table 2** Evaluation of the performance of hybrid KNN models *vs* the KNN models with the correlation coefficient (*r*), root mean square error (*RMSE*), mean absolute error (*MAE*), relative mean absolute error (R*MAE*) & Willmot’s Index (*WI*) between the forecasted and observed *DO* in the testing phase. Blue coloured raw indicates the optimum model for the standalone model, and red coloured indicates the best-performing hybrid model.

| **Model** | **r** | **WI** | **RMSE mg/l)** | **MAE (mg/l)** | **RMAE (%)** | **Performance** |
| --- | --- | --- | --- | --- | --- | --- |
| KNN_1_ | 0.372 | 0.445 | 0.581 | 0.475 | 13.594 | ↓ |
| KNN_3_ | 0.469 | 0.557 | 0.553 | 0.450 | 12.820 | ↑ |
| KNN_5_ | 0.588 | 0.652 | 0.513 | 0.413 | 11.730 | ↑ |
| KNN_7_ | 0.678 | 0.732 | 0.468 | 0.380 | 10.875 | ↑ |
| KNN_9_ | 0.683 | 0.749 | 0.464 | 0.375 | 10.687 | ↑ |
| KNN_14_ | 0.727 | 0.785 | 0.442 | 0.356 | 10.022 | ↑ |
| KNN_15_ | 0.713 | 0.781 | 0.447 | 0.361 | 10.220 | ↓ |
| KNN_17_ | 0.722 | 0.784 | 0.443 | 0.358 | 10.137 | ↑ |
| KNN_19_ | 0.710 | 0.780 | 0.447 | 0.362 | 10.213 | ↓ |
| KNN_22_ | 0.714 | 0.782 | 0.445 | 0.368 | 10.434 | ↑ |
| KNN_25_ | 0.701 | 0.773 | 0.450 | 0.365 | 10.418 | ↓ |
| KNN_26_ | 0.704 | 0.770 | 0.450 | 0.367 | 10.461 | ↑ |
| KNN_27_ | 0.714 | 0.785 | 0.442 | 0.360 | 10.279 | ↑ |
| **KNN_28_** | **0.786** | **0.830** | **0.400** | **0.329** | **9.3914** | **↑** |
| KNN_29_ | 0.783 | 0.834 | 0.399 | 0.325 | 9.2631 | ↓ |
| KNN_30_ | 0.783 | 0.834 | 0.399 | 0.326 | 9.2872 | ↑ |
| KNN-EMD | 0.819 | 0.865 | 0.370 | 0.305 | 8.770 | ↑ |
| KNN-EEMD | 0.813 | 0.845 | 0.384 | 0.326 | 9.300 | ↓ |
| **KNN-CEEMDAN** | **0.819** | **0.838** | **0.387** | **0.324** | **9.407** | ↑ |
| KNN-DWT | 0.692 | 0.770 | 0.453 | 0.358 | 10.509 | ↓ |
| KNN-MODWT | 0.708 | 0.734 | 0.464 | 0.378 | 10.748 | ↓ |

**Table 3** Evaluation of the performance of hybrid KRR models *vs* the KRR models with the correlation coefficient (*r*), root mean square error (*RMSE*), mean absolute error (*MAE*), relative mean absolute error (R*MAE*) & Willmot’s Index (*WI*) between the forecasted and observed *DO* in the testing phase. Blue coloured raw indicates the optimum model for the standalone model, and red coloured indicates the best-performing hybrid model.

| **Model** | **r** | **WI** | **RMSE mg/l)** | **MAE (mg/l)** | **RMAE (%)** | **Performance** |
| --- | --- | --- | --- | --- | --- | --- |
| KRR_4_ | 0.442 | 0.537 | 0.561 | 0.455 | 12.948 | ↓ |
| KRR_7_ | 0.542 | 0.607 | 0.527 | 0.434 | 12.438 | ↑ |
| KRR_10_ | 0.580 | 0.645 | 0.512 | 0.425 | 12.171 | ↑ |
| KRR_13_ | 0.596 | 0.659 | 0.505 | 0.420 | 12.060 | ↑ |
| KRR_15_ | 0.605 | 0.659 | 0.502 | 0.419 | 12.027 | ↑ |
| KRR_17_ | 0.603 | 0.654 | 0.503 | 0.419 | 12.054 | ↓ |
| KRR_18_ | 0.606 | 0.652 | 0.503 | 0.418 | 12.030 | ↑ |
| KRR_19_ | 0.611 | 0.654 | 0.502 | 0.420 | 12.065 | ↑ |
| KRR_20_ | 0.619 | 0.663 | 0.498 | 0.417 | 12.003 | ↑ |
| KRR_21_ | 0.623 | 0.663 | 0.497 | 0.416 | 11.988 | ↑ |
| KRR_22_ | 0.625 | 0.662 | 0.496 | 0.416 | 11.988 | ↑ |
| KRR_23_ | 0.627 | 0.663 | 0.496 | 0.415 | 11.976 | ↑ |
| KRR_24_ | 0.630 | 0.662 | 0.495 | 0.416 | 11.976 | ↑ |
| KRR_25_ | 0.633 | 0.663 | 0.495 | 0.414 | 11.948 | ↑ |
| KRR_26_ | 0.636 | 0.663 | 0.494 | 0.413 | 11.907 | ↑ |
| KRR_27_ | 0.638 | 0.666 | 0.493 | 0.411 | 11.868 | ↑ |
| **KRR_28_** | **0.781** | **0.795** | **0.417** | **0.346** | **9.940** | ↑ |
| KRR_29_ | 0.780 | 0.794 | 0.417 | 0.346 | 9.968 | ↓ |
| KRR_30_ | 0.779 | 0.791 | 0.419 | 0.348 | 10.011 | ↓ |
| KRR-EMD | 0.813 | 0.835 | 0.388 | 0.320 | 9.254 | ↑ |
| **KRR-EEMD** | **0.815** | **0.760** | **0.428** | **0.343** | **9.890** | ↑ |
| KRR-CEEMDAN | 0.778 | 0.744 | 0.448 | 0.367 | 10.422 | ↓ |
| KRR-DWT | 0.767 | 0.812 | 0.414 | 0.329 | 9.606 | ↑ |
| KRR-MODWT | 0.710 | 0.564 | 0.514 | 0.421 | 11.948 | ↓ |

**Table 4** Evaluation of the performance of hybrid MARS models *vs* the MARS models with the correlation coefficient (*r*), root mean square error (*RMSE*), mean absolute error (*MAE*), relative mean absolute error (R*MAE*) & Willmot’s Index (*WI*) between the forecasted and observed *DO* in the testing phase. Blue coloured raw indicates the optimum model for the standalone model, and red coloured indicates the best-performing hybrid model.

| **Model** | **r** | **WI** | **RMSE mg/l)** | **MAE (mg/l)** | **RMAE (%)** | **Performance** |
| --- | --- | --- | --- | --- | --- | --- |
| MARS_2_ | 0.475 | 0.547 | 0.550 | 0.462 | 13.308 | ↑ |
| MARS_3_ | 0.499 | 0.573 | 0.541 | 0.447 | 12.877 | ↑ |
| MARS_4_ | 0.547 | 0.628 | 0.523 | 0.424 | 12.218 | ↑ |
| MARS_5_ | 0.585 | 0.682 | 0.506 | 0.413 | 11.962 | ↑ |
| MARS_6_ | 0.576 | 0.673 | 0.509 | 0.416 | 12.058 | ↑ |
| MARS_7_ | 0.613 | 0.706 | 0.493 | 0.406 | 11.795 | ↑ |
| MARS_8_ | 0.629 | 0.734 | 0.484 | 0.401 | 11.611 | ↑ |
| MARS_9_ | 0.615 | 0.705 | 0.492 | 0.405 | 11.732 | ↓ |
| MARS_10_ | 0.602 | 0.691 | 0.498 | 0.412 | 11.965 | ↑ |
| MARS_11_ | 0.610 | 0.711 | 0.494 | 0.408 | 11.816 | ↑ |
| MARS_14_ | 0.639 | 0.739 | 0.479 | 0.390 | 11.294 | ↑ |
| MARS_17_ | 0.629 | 0.729 | 0.484 | 0.393 | 11.362 | ↑ |
| MARS_18_ | 0.665 | 0.759 | 0.466 | 0.377 | 10.942 | ↑ |
| MARS_19_ | 0.636 | 0.737 | 0.481 | 0.388 | 11.173 | ↓ |
| MARS_20_ | 0.639 | 0.755 | 0.479 | 0.380 | 10.914 | ↑ |
| MARS_21_ | 0.649 | 0.742 | 0.475 | 0.389 | 11.301 | ↑ |
| MARS_22_ | 0.653 | 0.766 | 0.471 | 0.376 | 10.830 | ↑ |
| MARS_23_ | 0.639 | 0.755 | 0.479 | 0.380 | 10.914 | ↓ |
| MARS_24_ | 0.645 | 0.731 | 0.478 | 0.391 | 11.336 | ↑ |
| MARS_25_ | 0.653 | 0.747 | 0.472 | 0.380 | 10.958 | ↑ |
| MARS_26_ | 0.649 | 0.748 | 0.474 | 0.373 | 10.774 | ↓ |
| MARS_27_ | 0.660 | 0.765 | 0.467 | 0.369 | 10.636 | ↑ |
| MARS_28_ | 0.820 | 0.888 | 0.357 | 0.273 | 7.890 | ↑ |
| **MARS_29_** | **0.824** | **0.895** | **0.353** | **0.277** | **7.978** | ↑ |
| MARS_30_ | 0.821 | 0.889 | 0.357 | 0.278 | 7.898 | ↓ |
| MARS-EMD | 0.933 | 0.9633 | 0.2262 | 0.187 | 5.405 | ↑ |
| MARS-EEMD | 0.923 | 0.9471 | 0.3157 | 0.230 | 6.869 | ↓ |
| MARS-CEEMDAN | 0.949 | 0.9711 | 0.1992 | 0.156 | 4.654 | ↑ |
| MARS-DWT | 0.908 | 0.9238 | 0.2889 | 0.227 | 6.497 | ↓ |
| **MARS-MODWT** | **0.981** | **0.9904** | **0.1205** | **0.089** | **2.467** | ↑ |

**Table 5** Evaluation of the performance of hybrid SVR models *vs* the SVR models with the correlation coefficient (*r*), root mean square error (*RMSE*), mean absolute error (*MAE*), relative mean absolute error (R*MAE*) & Willmot’s Index (*WI*) between the forecasted and observed *DO* in the testing phase. Blue coloured raw indicates the optimum model for the standalone model, and red coloured indicates the best-performing hybrid model.

| **Model** | **r** | **WI** | **RMSE mg/l)** | **MAE (mg/l)** | **RMAE (%)** | **Performance** |
| --- | --- | --- | --- | --- | --- | --- |
| SVR_1_ | 0.306 | 0.438 | 0.643 | 0.512 | 14.0379 |  |
| SVR _4_ | 0.600 | 0.695 | 0.526 | 0.392 | 11.0226 | ↑ |
| SVR _6_ | 0.678 | 0.767 | 0.484 | 0.368 | 10.3113 | ↑ |
| SVR _9_ | 0.711 | 0.799 | 0.461 | 0.352 | 9.7679 | ↑ |
| SVR _10_ | 0.739 | 0.819 | 0.446 | 0.336 | 9.1829 | ↑ |
| SVR _12_ | 0.863 | 0.914 | 0.334 | 0.222 | 5.9530 | ↑ |
| SVR _13_ | 0.858 | 0.887 | 0.359 | 0.278 | 7.5420 | ↓ |
| SVR _15_ | 0.867 | 0.893 | 0.351 | 0.263 | 7.0040 | ↑ |
| SVR _18_ | 0.865 | 0.889 | 0.357 | 0.264 | 6.8937 | ↓ |
| **SVR _20_** | **0.878** | **0.908** | **0.332** | **0.250** | **6.6523** | ↑ |
| SVR _21_ | 0.875 | 0.904 | 0.339 | 0.251 | 6.6035 | ↓ |
| SVR _22_ | 0.877 | 0.906 | 0.336 | 0.249 | 6.5828 | ↑ |
| SVR _23_ | 0.875 | 0.904 | 0.338 | 0.252 | 6.6531 | ↓ |
| SVR _24_ | 0.871 | 0.901 | 0.344 | 0.255 | 6.7203 | ↓ |
| SVR _25_ | 0.871 | 0.897 | 0.345 | 0.255 | 6.7091 | ↓ |
| SVR _26_ | 0.873 | 0.899 | 0.343 | 0.255 | 6.7265 | ↑ |
| SVR _27_ | 0.871 | 0.8982 | 0.345 | 0.259 | 6.8651 | ↓ |
| SVR _28_ | 0.868 | 0.9155 | 0.329 | 0.222 | 6.0046 | ↓ |
| SVR _29_ | 0.866 | 0.9148 | 0.331 | 0.223 | 6.0224 | ↓ |
| SVR _30_ | 0.863 | 0.9140 | 0.334 | 0.222 | 5.9530 | ↓ |
| SVR-EMD | 0.911 | 0.943 | 0.277 | 0.200 | 5.448 | ↑ |
| **SVR-EEMD** | **0.969** | **0.978** | **0.181** | **0.141** | **3.918** | **↑** |
| SVR-CEEMDAN | 0.971 | 0.983 | 0.161 | 0.121 | 3.364 | ↑ |
| SVR-DWT | 0.926 | 0.938 | 0.266 | 0.208 | 6.038 | ↓ |
| SVR-MODWT | 0.959 | 0.967 | 0.213 | 0.147 | 4.022 | ↑ |

**Table 6** Evaluation of the performance of hybrid RF models *vs* the RF models with the correlation coefficient (*r*), root mean square error (*RMSE*), mean absolute error (*MAE*), relative mean absolute error (R*MAE*) & Willmot’s Index (*WI*) between the forecasted and observed *DO* in the testing phase. Blue coloured raw indicates the optimum model for the standalone model, and red coloured indicates the best-performing hybrid model.

| **Model** | **r** | **WI** | **RMSE mg/l)** | **MAE (mg/l)** | **RMAE (%)** | **Performance** |
| --- | --- | --- | --- | --- | --- | --- |
| RF _1_ | 0.303 | 0.366 | 0.629 | 0.511 | 14.349 |  |
| RF _2_ | 0.610 | 0.636 | 0.533 | 0.438 | 12.592 | ↑ |
| RF _3_ | 0.648 | 0.659 | 0.520 | 0.427 | 12.205 | ↑ |
| RF _4_ | 0.647 | 0.672 | 0.517 | 0.416 | 11.842 | ↓ |
| RF _5_ | 0.658 | 0.721 | 0.501 | 0.405 | 11.602 | ↑ |
| RF _7_ | 0.666 | 0.733 | 0.496 | 0.407 | 11.708 | ↑ |
| RF _8_ | 0.671 | 0.740 | 0.492 | 0.404 | 11.594 | ↑ |
| RF _12_ | 0.703 | 0.752 | 0.478 | 0.391 | 11.131 | ↑ |
| RF _14_ | 0.704 | 0.762 | 0.475 | 0.377 | 10.720 | ↑ |
| **RF _19_** | **0.767** | **0.842** | **0.421** | **0.318** | **8.899** | ↑ |
| RF _20_ | 0.723 | 0.773 | 0.465 | 0.371 | 10.574 | ↓ |
| RF _24_ | 0.722 | 0.769 | 0.467 | 0.372 | 10.600 | ↓ |
| RF _25_ | 0.722 | 0.769 | 0.467 | 0.372 | 10.599 | ↓ |
| RF _26_ | 0.720 | 0.767 | 0.468 | 0.374 | 10.645 | ↓ |
| RF _29_ | 0.766 | 0.841 | 0.423 | 0.319 | 8.896 | ↑ |
| RF _30_ | 0.765 | 0.841 | 0.424 | 0.319 | 8.899 | ↓ |
| RF-EMD | 0.753 | 0.754 | 0.472 | 0.370 | 10.322 | ↓ |
| RF-EEMD | 0.784 | 0.821 | 0.425 | 0.348 | 9.825 | ↑ |
| RF-CEEMDAN | 0.795 | 0.834 | 0.399 | 0.326 | 9.746 | ↑ |
| RF-DWT | 0.765 | 0.841 | 0.424 | 0.319 | 8.899 | ↓ |
| **RF-MODWT** | **0.837** | **0.784** | **0.416** | **0.333** | **9.821** | **↑** |
